# Functions, structure, and read-through alternative splicing of feline APOBEC3 genes

**DOI:** 10.1186/gb-2008-9-3-r48

**Published:** 2008-03-03

**Authors:** Carsten Münk, Thomas Beck, Jörg Zielonka, Agnes Hotz-Wagenblatt, Sarah Chareza, Marion Battenberg, Jens Thielebein, Klaus Cichutek, Ignacio G Bravo, Stephen J O'Brien, Martin Lochelt, Naoya Yuhki

**Affiliations:** 1 Division of Medical Biotechnology, Paul-Ehrlich-Institut, 63225 Langen, Germany; 2 SAIC-Frederick, Inc., NCI-Frederick, Laboratory of Genomic Diversity, Frederick, MD 21702-1201, USA; 3 Department of Molecular Biophysics, Research Program Structural and Functional Genomics, German Cancer Research Center, 69120 Heidelberg, Germany; 4 Department of Genome Modifications and Carcinogenesis, Research Program Infection and Cancer, German Cancer Research Center, Heidelberg, Germany; 5 Institute of Agricultural and Nutritional Sciences, Martin-Luther-University Halle-Wittenberg, 06108 Halle, Germany; 6 Institute for Evolution and Biodiversity, Westfälische Wilhems University Münster, 48143 Münster, Germany; 7 Laboratory of Genomic Diversity, NCI at Frederick, Frederick, MD 21702-1201, USA

## Abstract

**Background:**

Over the past years a variety of host restriction genes have been identified in human and mammals that modulate retrovirus infectivity, replication, assembly, and/or cross-species transmission. Among these host-encoded restriction factors, the APOBEC3 (A3; apolipoprotein B mRNA-editing catalytic polypeptide 3) proteins are potent inhibitors of retroviruses and retrotransposons. While primates encode seven of these genes (A3A to A3H), rodents carry only a single A3 gene.

**Results:**

Here we identified and characterized several A3 genes in the genome of domestic cat (*Felis catus*) by analyzing the genomic A3 locus. The cat genome presents one A3H gene and three very similar A3C genes (a-c), probably generated after two consecutive gene duplications. In addition to these four one-domain A3 proteins, a fifth A3, designated A3CH, is expressed by read-through alternative splicing. Specific feline A3 proteins selectively inactivated only defined genera of feline retroviruses: Bet-deficient feline foamy virus was mainly inactivated by feA3Ca, feA3Cb, and feA3Cc, while feA3H and feA3CH were only weakly active. The infectivity of Vif-deficient feline immunodeficiency virus and feline leukemia virus was reduced only by feA3H and feA3CH, but not by any of the feA3Cs. Within Felidae, A3C sequences show significant adaptive selection, but unexpectedly, the A3H sequences present more sites that are under purifying selection.

**Conclusion:**

Our data support a complex evolutionary history of expansion, divergence, selection and individual extinction of antiviral A3 genes that parallels the early evolution of Placentalia, becoming more intricate in taxa in which the arms race between host and retroviruses is harsher.

## Abstract 2

APOBEC3 (A3, Apolipoprotein B mRNA-editing catalytic polypeptide 3) genes in the genome of domestic cat (Felis catus) were identified and characterized

## Background

The domestic cat (*Felis catus*) is an established animal model for studies of the brain, genetics, pharmacology, and nutrition [1]. In addition, the cat serves as a model for viral infectious diseases. For instance, since feline immunodeficiency virus (FIV) shares many features in common with human immunodeficiency virus (HIV), FIV-infected cats serve as an important model for HIV/AIDS, for example, with respect to therapy, vaccination and pathogenesis [2]. In addition, two other exogenous retroviruses are prevalent in cats, with very different outcomes of infection. Feline leukemia virus (FeLV) is a serious oncogenic pathogen of cats [3] whereas feline foamy virus (FFV) has not been firmly linked to any disease [4] and shows potential as a gene transfer vehicle for cats [5]. FIV is endemic to at least 21 free ranging Felidae species, including lion, cheetah, and puma as well as domestic cat [6], while the prevalence of other feline viruses is less characterized. Although molecular and genetic features of these feline retroviruses have been unraveled over the past years, studies on the contribution of host genes in permissiveness towards virus replication and especially in actively restricting virus multiplication, determining disease, and influencing spread and transmission are only now becoming possible due to new achievements in genomics. Recently, the lightly covered whole genome shotgun (WGS) sequences of the domestic cat (1.9× genome coverage) were assembled and annotated based on the comparison with conserved sequence blocks of the genome sequences of human and dog [7]. The detailed upcoming 7× WGS sequence and analysis of the feline genome will provide an important mammalian comparative genome sequence relative to primates (human and chimpanzee), rodents (mouse and rat), and carnivores (cat and dog) and will likely provide new insights into disease inheritance and the relationship between genetic background of the host and infectious diseases.

The APOBEC3 (A3; for apolipoprotein B mRNA-editing catalytic polypeptide 3) genes are of particular interest because they form part of the intrinsic immunity against retroviruses (for a review see [8]), are under a high adaptive selection [9], and might have undergone a relatively recent unique evolutionary expansion in primates [10]. In humans, A3F and A3G specifically are capable of terminally editing HIV-1 by deamination of cytidine to uracil during reverse transcription in addition to other, still ill-defined antiviral activities [11]. However, the virion infectivity factor (Vif) of HIV actively counteracts this host-mediated restriction [12-16]. The interaction between Vif and A3 proteins is species-specific and may thus limit cross-species virus transmission [17]. Similar editing has been implicated in the replication of a number of viruses, including simian immunodeficiency virus (SIV), FFV, FIV and hepatitis B virus [18-21]. While foamy retroviruses also utilize an accessory viral protein (Bet) to counteract A3 inactivation, other viruses like human T-cell leukemia virus have evolved *vif*-independent mechanisms to evade A3-mediated restriction, underpinning the importance of this host restriction [21-23].

Our objective was to identify and characterize A3 genes in the feline genome and compare them to those in the human and dog genomes. Fosmid clones used for the 1.9× WGS cat genome project and the accompanying data were organized into a database that could be used for targeted sequencing of regions underrepresented in the 1.9× genome sequence of the cat. We have used this resource to characterize the feline A3 region and to infer its evolutionary history. Our results reveal that, within Felinae, the A3 locus underwent a unique triplication of the A3C gene, whereas the A3H gene exists as a single copy. In addition, we found a gene read-through generating a double-domain A3CH protein. APOBEC3 proteins of the cat are active inhibitors of various feline retroviruses and show differential target specificity.

## Results

Recently we described an antiviral cytidine deaminase of the A3 family in cells of the domestic cat [21]. Feline A3 (feA3) was found to be an active inhibitor of *bet*-deficient FFV [21] and SIV (data not shown and see below), but failed to show antiviral activity against wild-type or Δ*vif *FIV (data not shown and see below). However, the presence of a *vif *gene in the FIV genome, assumed to counteract the anti-viral activity of A3 proteins, strongly argues for additional feline APOBEC3s in the cat. This prompted us to search the genome of *F. catus *more thoroughly for A3 genes. Initial attempts to clone cat A3 cDNAs by a combination of PCR and 5' and 3' rapid amplification of cDNA ends (RACE) detected, in addition to feA3, at least two more A3-related RNAs in the feline cell line CrFK [24].

### Genomic organization of the feline APOBEC3 locus

**Figure 1:**
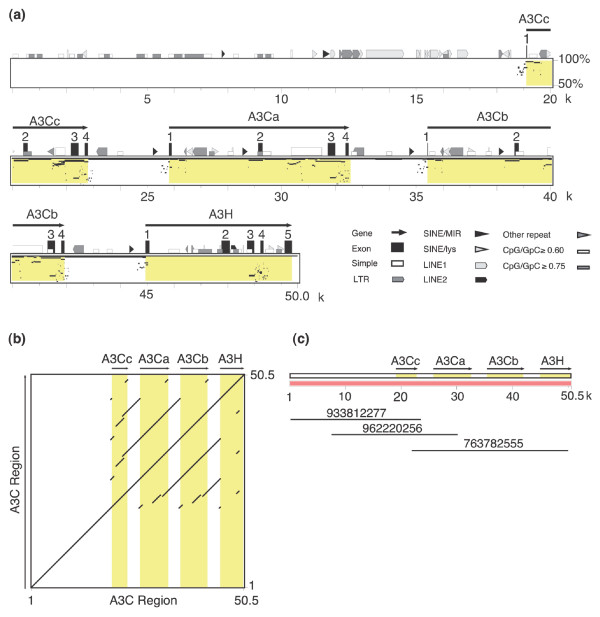
Gene organization of the feline APOBEC3 region. **(a) **Shown is a Pipmaker analysis of the 50 kb nucleotide sequence of the APOBEC3 region showing the intron/exon organization of the four identified feline A3 genes (A3Cc, A3Ca, A3Cb and A3H) and annotation of repetitive elements (see inset for key: Simple, simple repeat sequence poly(dT-dG).poly(dC-dA); LTR, long terminal repeat retrotransposons; SINE, short interspersed elements; SINE/MIR, MIRs are tRNA-derived SINEs that amplified before the mammalian radiation; SINE/lys, tRNA-lys-derived SINE; LINE1, long interspersed element 1; LINE 2, long interspersed element-2; CpG/GpC ratios are indicated). **(b) **Organization and gene content of the fosmids used for nucleotide sequencing. **(c) **Self-dotplot of the percent identities of the A3C region showing the high degree of sequence identity between A3Cc, A3Ca, and A3Cb.

Comparative genomic analysis has shown that the genome of the domestic cat contains gene sequences orthologous to AICDA (activation-induced cytidine deaminase; also known as AID) and APOBEC1, 2, 3 and 4 in that they map to syntenic chromosomal regions of human and dog. The chromosomal localizations of APOBEC1, 2, 3 and 4, were determined on cat chromosomes B4, B2, B4, and F1, respectively. To further identify and characterize A3 genes in the annotated Abyssinian cat genome sequence, we studied fosmids that had been end-sequenced as part of the 1.9× domestic cat genome project from Agencourt Bioscience Corporation. To establish a web-based fosmid cloning system, the 1,806 fosmid 384-well plates were stored in assigned locations. A fosmid database of 1,288,606 fosmid clones, sequence-trace IDs, plate and well IDs, and freezer location IDs was generated and linked to the GARFIELD browser and the National Center for Biotechnology Information (NCBI) trace IDs. In this system, fosmid cloning is achieved by using potential orthologues (that is, human, mouse, dog or yeast) of genes of interest and searching for fosmid trace IDs by gene ID/symbol in the GARFIELD browser or by discontinuous MEGABLAST of orthologous sequences to *F. catus *WGS at the NCBI BLAST site. With the trace ID, the fosmid freezer location ID can be retrieved from the fosmid database. We have tested 704 fosmids and could identify with a 99% accuracy 616 of them (87.5%), as confirmed by fosmid end-sequencing. Using this system, we selected a total of seven fosmids that were predicted to encompass A3 genes and three clones were subsequently analyzed by nucleotide sequencing (Figure 1).

**Figure 2:**
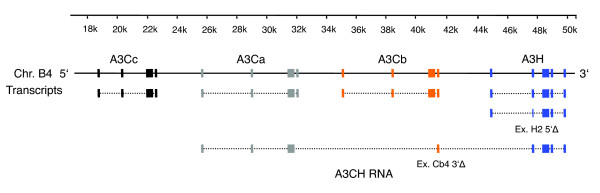
Representation of the feline APOBEC3 genomic region, portraying the detected A3 transcripts. Transcripts with translated exon (rectangles) and spliced-out introns (dotted lines) are indicated. Please note that the transcript for A3H comes in two versions: with complete exon 2 and further spliced exon 2, resulting in 5' truncation (Ex. H2 5'Δ). The mRNA for A3CH includes exon 4 of A3Cb in an additionally spliced version, 3' truncating the sequence one nucleotide before the stop codon (Ex. Cb4 3'Δ).

Figure 1a shows a percent identity alignment of the 50 kb A3 region sequenced aligned to itself. Gene modeling studies using the predicted nucleotide and amino acid sequences of cat A3 and A3H cDNAs and the programs Spidey [25] and Genewise [26] demonstrated the presence of three feline A3C genes designated A3Ca (identical to A3C cDNA [21]), A3Cb and A3Cc and a single A3H gene arrayed in a head-to-tail formation spanning 32 kb of the 50 kb region sequenced (Figure 1a,b). The A3C genes each consist of four exons with coding sequences that span 3,693, 6,457 and 6,498 bp for A3Cc, A3Ca, and A3Cb, respectively, whereas A3H contains one 5' untranslated exon followed by four coding exons that span 2,237 bp (Figures 1 and 2). Consensus splice acceptor sites were observed for exons 2 to 4 in the three A3C genes and exons 2 to 5 in the A3H gene. Consensus splice donor sites were observed for exons 1 to 4 in A3H and in all four coding exons of the three A3C genes. Interestingly, splicing at the splice donor sites of exon 4 (bold) in all A3C genes eliminates the overlapping termination codon (underlined) of the feA3 cDNA (CTT **AGG ****T**GA), allowing the generation of chimeric read-through transcripts. Consensus polyadenylation signals (AATAAA) were observed at positions downstream of exon 4 for all three A3C genes - A3Ca (positions 32,505, 33,376 and 33,444), A3Cb (positions 42,083, 42,954 and 43,022), and A3Cc (positions 22,960 and 23,831) - and A3H (position 50,319).

**Figure 3:**
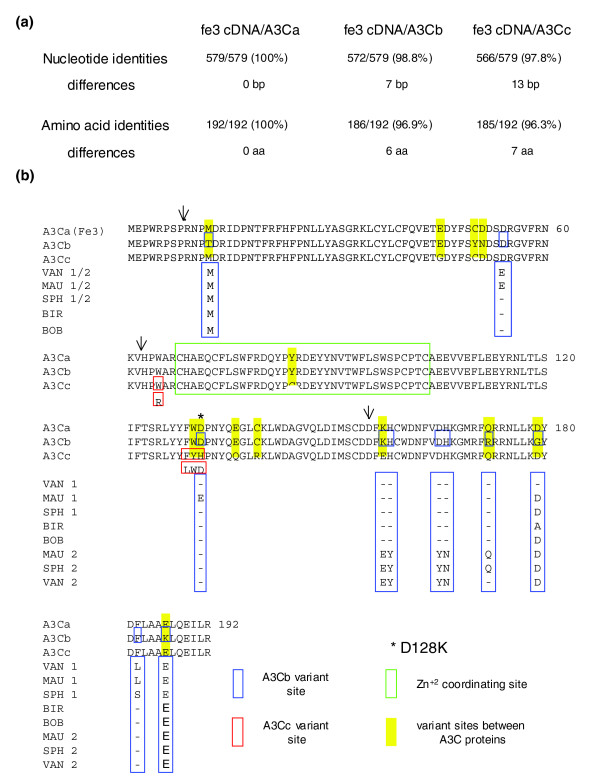
Comparison of the nucleotide coding and amino acid sequences of the feline A3C genes. **(a) **Pairwise comparison of the domestic cat A3 cDNA to the predicted A3Ca, A3Cb and A3Cc genomic coding sequences and the predicted amino acid sequences. **(b) **Amino acid sequence alignment of A3C cDNA and the predicted proteins for A3C genes. Highlighted in yellow are amino acid residues different between the A3Cs based on the genomic sequence, whereas amino acid sites displaying non-synonymous amino acid substitutions are boxed in blue and red for A3Cb and A3Cc, respectively, as identified by SNP genotyping of eight domestic cat breeds for exons 2-4 of A3Ca, A3Cb and A3Cc (for more details see Table 4 in Additional data file 2). Arrows indicate exonic junctions. Below the alignments, variant amino acids are boxed in red for A3Cc (for example, W65R) and blue for A3Cb with respect to the breed from which they were identified: Turkish van (VAN), Egyptian mau (MAU), Sphynx (SPH), Birman (BIR) and Japanese bobtail (BOB). A dash indicates the amino acid is identical to genomic sequence. Numbers adjacent to breed identifiers refer to alleles 1 and 2 identified by clonal sequence analysis of the PCR products that are in phase for exons 3 and 4, but not for exon 2 (1/2). The residue corresponding to functionally significant amino acid replacement identified in human A3G (D128K) is highlighted by an asterisk (see text).

**Figure 4:**
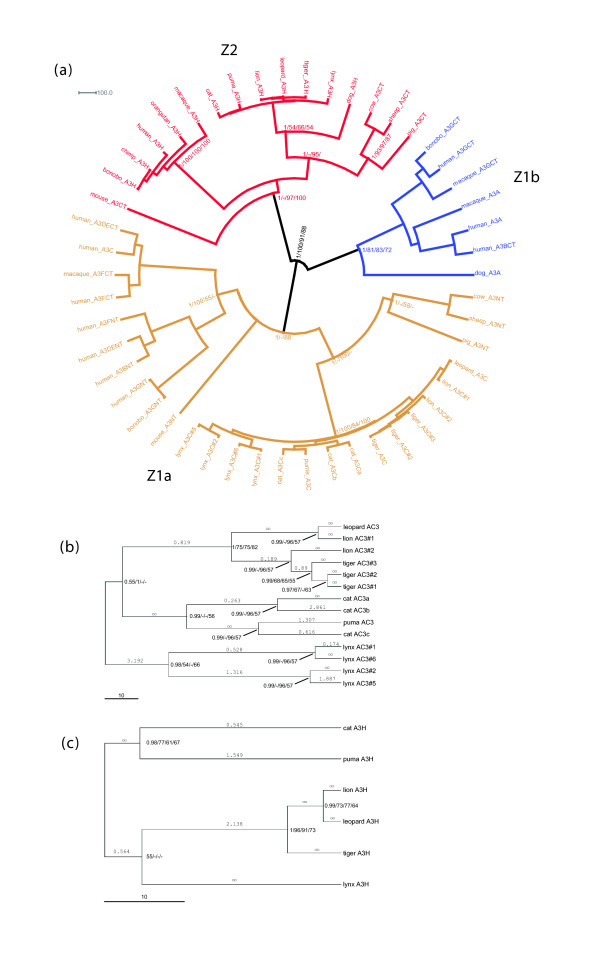
Phylogenetic analyses of the feline A3C and A3H genes. **(a) **Maximum clade credibility tree obtained after Bayesian phylogenetic inference with BEAST for the three large clusters of APOBEC3 sequences: A3A, A3C and A3H. Domains in two-domain proteins were split and analyzed separately, their position in the original sequence indicated as CT or NT, for carboxy-terminal or amino-terminal, respectively. Values in the nodes indicate corresponding support, as follows: Bayesian posterior probability/maximum likelihood (percentage after 500 cycles bootstrap)/distance analysis (percentage after 1,000 cycle bootstrap) parsimony analysis (percentage after 1,000 cycles bootstrap). The scale bar is given in substitutions per site. The domains within the A3 proteins can be divided into three groups of related proteins: A3H (Z2), A3A (Z1B) and A3C (Z1A). **(b,c) **Zoom-in on the maximum clade credibility tree obtained after Bayesian phylogenetic inference with BEAST, focusing on the Felinae APOBEC3C sequences (b) and APOBEC3H (c) sequences. Values in the nodes indicate corresponding support, as in the main tree in (a). The scale bar is given in substitutions per site. Figures above the branches indicate Ka/Ks ratios, calculated using Diverge. In some instances, zero synonymous substitutions lead to an apparent Ka/Ks ratio of infinity.

The initially identified cDNA of feA3 (A3Ca) and the coding sequences of the genes A3Cb and A3Cc show 97.6% and 98.9% identical nucleotides, respectively, and 96.3-96.5% identical amino acids to each other. The predicted proteins of A3Cb and A3Cc differ in six or seven amino acids from feA3 (A3Ca; Figure 3; Figure S2 in Additional data file 3). The feline A3C genes show high overall similarity to human A3C with 43.3-43.8% identical amino acids (Figure S2 in Additional data file 3). In addition to the high degree of sequence identity between the coding sequences of the three cat A3C genes, the pattern of repetitive elements, especially in intron 1 of A3Ca and A3Cb (Figure 1A), and self dotplot analyses (Figure 1c) suggested significant sequence identity in noncoding regions of these highly related genes. Supplementary Table 1 in Additional data file 2 shows the size of each intron and the pairwise percent identities between the introns of the three genes: the introns of A3Ca and A3Cb have a high degree of nucleotide sequence identity (98-99%) across all three introns whereas A3Cc shows a lower degree of sequence identity to either A3Ca or A3Cb (67-96%), depending on the size of the intron. Based on the very high similarity of the A3C genes, two gene duplications in rather recent evolutionary times seem to be highly likely. The first duplication yielded A3Cc and an A3Ca/b progenitor gene. A3Ca/b subsequently duplicated again, resulting in A3Ca and A3Cb. As expected, the cat A3C genes have a more distant relationship to the human A3C group, the feline A3H clusters with the dog A3H gene, but the dog A3A is only distantly related to human A3A (Figure 4a). Double-domain APOBEC3 genes structurally analogous to human A3F or A3G have not been found in the genomes of either cat or dog.

### Expression of feline APOBEC3 genes

**Figure 5:**
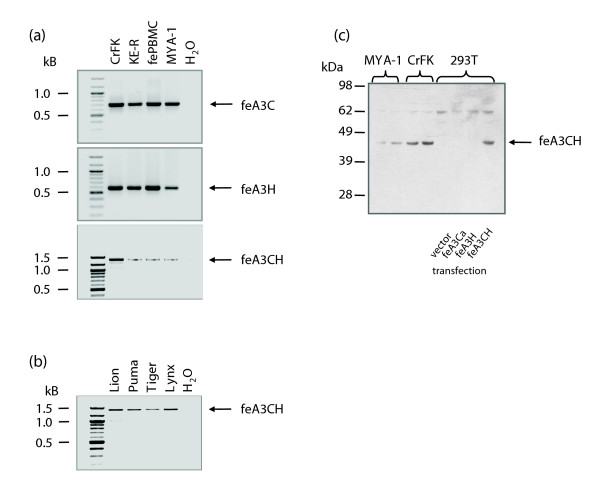
Expression analysis of feline A3C, A3H and A3CH. **(a,b) **Analysis of expression of feline A3C, A3H and A3CH by RT-PCR of total RNA from feline cell lines (CrFK, MYA-1, KE-R) and feline PBMCs (a) and expression of A3CH in PBMCs of lion, puma, Sumatra-tiger (tiger), and lynx (b). H_2_O indicates PCRs using primers specific for the A3s without template cDNA added. **(c) **Analysis of expression of cat A3CH by immunoblot using rabbit serum against the sequence flanked by the C- and H-domains in cat A3C (linker) using 293T cells transfected with A3 expression plasmid or empty vector as indicated and CrFK and MyA-1 cells (two independent cultures each).

Initially, we applied 3' RACE assays using the A3Ca sequence in order to clone additional feline A3 cDNAs. We detected the single-domain A3H and a cDNA composed of the fused open reading frames of A3C and A3H, designated A3CH [24]. A closer inspection of the sequence of A3CH revealed that the transcript is encoded by exons 1-3 of A3Ca, the complete coding sequence of exon 4 of A3Cb and exons 2-5 of A3H (Figure 2). Importantly, the consensus splice donor of exon 4 of A3Cb, located only one nucleotide 5' of the stop codon TGA, is used for in-frame splicing to A3H exons 2-5. The double-domain A3CH RNA was found in three tested cell lines (CrFK, MYA-1, KE-R) and also in feline peripheral blood mononuclear cells (PBMCs; Figure 5a). In 20 cloned PCR products from independent reverse-transcriptase (RT)-PCR reactions using RNA from CrFK, MYA-1 and PBMCs, the A3CH cDNAs were always exactly as described above (exons 1-3 of A3Ca, the 3' truncated exon 4 of A3Cb and exons 2-5 of A3H). In no case did we observe sequence variation in the A3CH mRNA, for example, by contribution of other A3C exons. We used diagnostic PCR primers to analyze the expression of A3Ca, A3Cb, A3Cc, and A3H in total RNA of feline PBMCs (of two cats of unknown pedigree) and cell lines (CrFK, KE-R, MYA-1). About half of the mRNAs from the activated feline PBMCs corresponded to A3Ca (22 of 40 clones) and approximately 17% were identical with A3Cb (7 of 40 clones) as determined by RT-PCR allowing detection of all three A3Cs. The remaining PCR products of A3C cDNAs represented additional variants, designated A3Cx and A3Cy, each containing six amino acid differences relative to A3Ca (Figure S1 in Additional data file 3), indicating further genetic allelic variation in cats. Sequence-based genotyping by direct PCR of genomic DNA using locus specific primers for exons 2-4 from eight domestic cat breeds resulted in finding zero, thirteen, and four non-synonymous substitutions and zero, one, and two synonymous substitutions in A3Ca, A3Cb and A3Cc genes, respectively (Figure 3b; details in supplementary Table 4 in Additional data file 2). MYA-1 cells expressed A3Ca, A3Cb and A3Cc genes (15, 5 and 1 clone out of 21, respectively), but CrFK and KE-R cells expressed only A3Ca (10 of 10 clones for each). Feline A3H was detected in all analyzed cell lines and PBMCs (Figure 5a). Interestingly, the transcript for A3H seems to be subject to alternative splicing, since we consistently detected an additional variant containing a 5' truncated exon 2, generating a cDNA with a 149 nucleotide shorter 5' untranslated region (Figure 2).

In order to determine whether the different A3 proteins are present in feline CrFK and MYA-1 cells, immunoblot analyses using antisera directed against cat A3C and A3H as well as a serum directed against the A3CH-specific sequence flanked by the C- and H-domains in A3CH (linker) were employed. In extracts from CrFK and MYA-1 cells the anti-linker serum detected a protein band that clearly co-migrated with A3CH expressed from plasmid pcfeA3CH in transfected 293T cells (Figure 5c). The C- and H-domain-specific antisera detected the corresponding A3C and A3H proteins in CrFK cells while only after over-exposition of the immunoblot was the A3CH protein detectable with these sera (data not shown). This detection pattern may reflect low-level expression of A3CH or may indicate that the corresponding epitopes are masked in the two-domain A3CH protein.

To search for transcription factor binding sites that might regulate A3 expression in the domestic cat A3 gene cluster, we first aligned the upstream 1.1 kb, including 100 bp of the predicted exon 1 for each gene A3Ca, A3Cb, A3Cc, and A3H using ClustalW. This analysis showed considerable sequence similarity in the proximal 5' flanking sequences of all four A3 genes, with A3Cc the most divergent (Figure S3 in Additional data file 3). Using MEME to search for conserved sequence elements in a set of DNA sequences using an expectation-maximization algorithm, we detected two highly conserved 50 bp sequence motifs between all four promoter regions, one located flanking the putative transcription start site and the other approximately 200 bp upstream [27,28]. Individual 5' flanking sequences were analyzed using the Match program, which uses a library of nucleotide weight matrices from the TRANSFAC6.0 database for transcription factor binding sites [29]. The first 50 bp motif contains putative transcription factor binding sites for HNF-4 and Elk-1 as well as a site reportedly present in all phenobarbital-inducible promoters 30 bp upstream of the transcriptional start site. No obvious TATA or CAAT boxes were identified, similar to the human A3 region [30]. The second site (200 bp upstream of the start site) includes Octamer and Evi-1 transcription factor binding sites, which are associated with transcription in hematopoietic cell lineages. Further 5', the sequences and predicted transcription factor binding sites of A3Ca, A3Cb and A3H are relatively well conserved whereas A3Cc is divergent, suggesting that A3Cc has a unique transcription profile as indicated in our RT-PCR expression studies. Another approach to identify transcription factor binding sites, ModelInspector uses a library of experimentally verified promoter modules or models that consist of paired transcription factor binding sites, orientation, order and distance. Using this method, we identified four paired transcription factor binding sites shared between one or more of the feline A3 promoters and that of human A3G [31], including two ETS-SP1 (A3Ca, A3Cb, A3Cc and A3H), IKRS-AP2 (A3H), and NFκB paired with either CEBP (A3Ca and A3Cb), RBPF (A3Ca, A3Cb and A3Cc) or STAT (A3Ca, A3Cb and A3H). Future studies are required to demonstrate the potency of these elements.

### Diversity of APOBEC3 in the family Felidae

It was demonstrated that primate A3 genes are under a strong positive selection predating modern lentiviruses [9,32,33]. Currently, it is not known whether the rapid adaptive selection of A3 genes is unique to primates or represents rather a general feature of Placentalia. To gain further insight into this question, we analyzed A3 sequences of additional Felidae species. We cloned the orthologous cDNAs of A3C and A3H from activated PBMCs of lion (*Panthera leo bleyenberghi*), two tiger subspecies (*Panthera tigris sumatrae *and *Panthera tigris corbetti*), leopard (*Panthera pardus japonensis*), lynx (*Lynx lynx*) and puma (*Puma concolor*). Together with *F. catus*, this collection comprises four of the eight extant lineages within Felidae [34]. We characterized two to six transcripts for A3C and A3H of each species, one animal per species. The phylogenetic relationships and identities to the domestic cat A3 genes are shown in Figure 4b,c, and supplementary Tables 5 and 6 in Additional data file 2. In lynx, lion and tiger, the cDNAs for A3C depicted some degree of intra-species genetic variability and all variants were included in our analysis. In three of six A3C isolates of Sumatra-tiger and both Indochina-tiger cDNAs, the sequence encoded a lysine at position 185, while in the three other clones of Sumatra-tiger a glutamate was encoded. No further diversity in A3C-cDNAs of Sumatra-tiger and Indochina-tiger was found. We detected only a single type of A3H transcript in each of the above-mentioned felid species. In Indochina-tiger A3H, we found a polymorphism encoding either an arginine or a lysine at amino acid position 65, whereas in A3H cDNAs of Sumatra-tiger, only K65 was seen. The A3CH transcript was also detected in cDNA preparations of lion, puma, Sumatra-tiger and lynx (leopard was not analyzed) (Figure 5b).

Comparing non-synonymous substitution rates (Ka) and synonymous substitution rates (Ks) within the alignment of the A3C and A3H cDNA sequences, several Ka/Ks ratios were above 1, indicating positive selection among the A3C sequences (Table 2 in Additional data file 2) and the A3H sequences (Table 3 in Additional data file 2) of the different felids. Because extreme Ka/Ks ratios below or above 1 may appear when only few residues are under positive or purifying selection, we used the sliding window approach to determine whether defined regions of the A3 proteins are under any type of selection. The results in Figure S4 in Additional data file 3 show that comparison of feline A3s to the corresponding human A3s do not show clear positive or negative selection as expected due to the evolutionary divergence. In contrast, positive selection of cat, tiger, lion and leopard A3Cs peaks around 200 bp (at the start of the Zn^2+^-coordinating domain) while comparison with lynx and puma A3Cs reveal different sites under positive selection. In the case of A3H the sliding window comparison was not meaningful because the small number of substitutions led to many infinity values due to Ks = 0. Therefore, the trees of the A3C and A3H genes (Figure 4b,c) were further tested for the presence of selection among amino acid sites using the Phylogenetic Analysis by Maximum Likelihood (PAML) program version 3.15 [35,36]. Evaluating the difference of the maximum likelihood values for the trees calculated with different evolutionary models, a probability estimate for positive selection can be deduced. In the case of A3H the difference is not statistically significant (*P *= 0.4; Additional data files 1 and 2), but in model 2, which allows for three different ω values (ω = 1 means neutral evolution, ω < 1 purifying selection, ω > 1 positive selection), 71% of A3H are summarized with ω = 0, supporting purifying selection as the simplest evolutionary model. In contrast, positive selection can be found for several residues for A3C sequences (*P *< 0.0001; 15% of A3C are summarized with ω = 7.2 under model 2). Comparable results were obtained when using the webserver Selecton version 2.2 [37,38] for cat A3Ca and cat A3Cc with the alignment of A3C with all felid species and for cat A3H using the alignment of A3H sequences (data not shown).

### The diverse feline APOBEC3s differentially inhibit feline retroviruses

**Figure 6:**
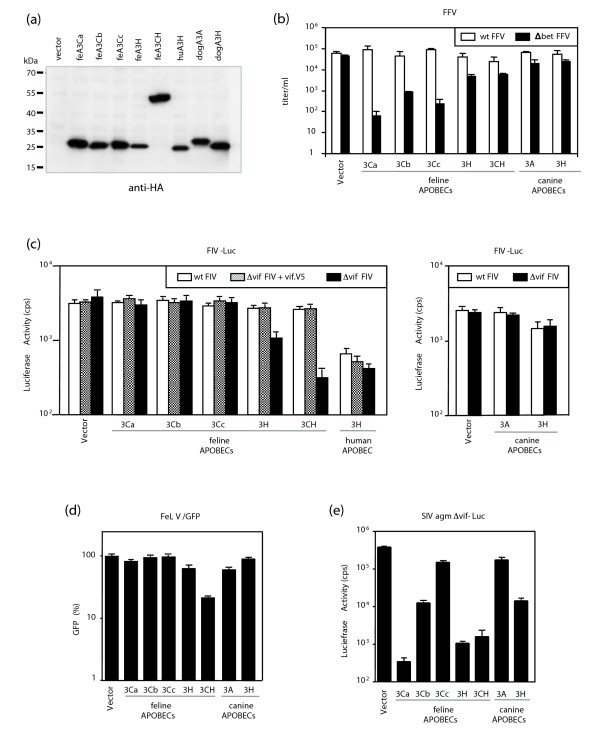
Cat A3 proteins selectively inhibit the infectivity of different retroviruses. **(a) **A3 expression in the transfected 293T cells was detected by immunoblotting with anti-HA monoclonal antibody. **(b-e) **Wild-type (wt) or Δ*bet *FFV wild type (b), wild-type FIV-Luc, Δvif FIV-Luc + Vif expression plasmid (vif.V5) and Δ*vif *FIV luciferase reporter vector particles (c), FeLV/GFP (d), and Δ*vif *SIVagm luciferase viruses (e) were produced in 293T cells in the presence or absence of the indicated APOBEC3s.

In a recent study we showed that cat A3Ca is a potent inhibitor of *bet*-deficient FFV (FFVΔ*bet*) [21]. We were interested to extend this finding and tested A3Ca, A3Cb, A3Cc, A3H and A3CH as well as dog A3A and A3H with viral reporter systems for FFV, FIV and FeLV. To monitor the activity of the A3s, plasmids expressing hemagglutinin (HA)-tagged versions of A3 were used. All A3 proteins could be detected in immunoblots; cat A3Ca, A3Cb, A3Cc and A3CH were comparably expressed, and the expression of cat and human A3H was reduced three- to five-fold (Figure 6a).

The effect of A3 co-expression on wild-type and Bet-deficient FFV was studied after transfection of 293T cells. For this purpose, the infectivity of FFV titers was determined two days after transfection by using FeFAB reporter cells [39]. Cotransfection of A3Ca did not reduce the wild-type FFV titer, whereas a 700-fold reduction in titer was detected with the Bet-deficient FFV (Figure 6b), as described previously [21]. Quite similarly, A3Cb and A3Cc did not inhibit wild-type FFV but reduced the titer of Δ*bet *FFV by 200- and 70-fold, respectively. Feline A3H and A3CH showed a comparable low antiviral activity and reduced Bet-deficient but not wild-type FFV to a much lower degree. Dog A3A and A3H did not inhibit the infectivity of Δ*bet *FFV or wild-type FFV. To assess the antiviral activity of the cat A3s on FIV, vesicular stomatitis virus-G protein (VSV-G) pseudotyped wild-type FIV-luciferase (FIV-Luc), Δ*vif *FIV-Luc and Δ*vif *FIV-Luc cotransfected with Vif expression plasmid (pcFIV.Vif-V5) reporter vectors were generated in 293T cells in the presence of A3 expression plasmids. Equal amounts of particles were used for transduction experiments. The results depicted in Figure 6c show that only two of the five cat A3 proteins are inhibitors of FIVΔ*vif*-mediated gene transfer: feline A3H and A3CH reduced the infectivity by five- and ten-fold, respectively, similar to the human A3H. Feline A3Ca, A3Cb or A3Cc and dog A3A expression plasmids did not reduce infectivity of wild-type or Δ*vif *FIV. In contrast, dog A3H showed antiviral activity against wild-type and Δ*vif *FIV, causing a three-fold reduction. We recently showed that the inactivation of Δ*bet *FFV and HIV-1 by feline A3s was attributable to cytidine deamination of viral reverse transcripts [21]. The suppression of Δ*vif *FIV by feline A3H and A3CH also correlates with a significant increased G→A mutation rate in the viral genomes (Figure S5a,b in Additional data file 3): cotransfection of feA3H or feA3CH resulted in 1.61% and 1.31% G→A substitutions, respectively. Viral genomes of Δ*vif *FIV derived from transfections omitting an A3 expression plasmid showed no G→A editing; using feA3Ca, feA3Cb or feA3Cc expression plasmids, only 0.1% G→A exchanges were detectable at most. These data highly correlate with the inhibitory activity detected in the infectivity studies. The presence of Vif protein inhibited the genome editing nearly completely (Figure S5 in Additional data file 3). The sequence context of the majority of the G→A exchanges in the viral genomes derived from co-expressing feA3H and feA3CH showed no clear preference for a dinucleotide: feA3H induced 17% GG→AG, 35% GA→AA and 42% GC→AC exchanges in the positive strand of the DNA. The editing context of the A3CH showed 28% GG→AG changes, 39% GA→AG mutations, and 28% GC→AC changes. Both A3s edited in 5-6% GT→AT dinucleotides (Figure S5c in Additional data file 3). Interestingly, in the FIV system, the more antiviral A3CH generated slightly lower numbers of mutations than the less antiviral A3H (Figure S5a,b,d in Additional data file 3). This result could point either to additional and unknown activities of A3 proteins or to differences between the degradation kinetics of uracil-containing DNAs.

To analyze the impact of cat A3 proteins on the infectivity of FeLV, we used a molecular clone of FeLV subgroup A (p61E-FeLV). Reporter particles were generated by co-transfection of the p61E-FeLV packaging construct, a murine leukemia virus (MLV)-based green fluorescent protein (GFP)-reporter genome, a VSV-G pseudotyping plasmid and the different A3 expression plasmids. The FeLV/GFP virions were normalized for RT activity and used for infection of 293T cells. The GFP expression pattern of the inoculated cells demonstrated that cat A3Cs and dog A3H did not reduce the infectivity of FeLV/GFP (Figure 6d). Cat A3H and dog A3A had a marginal effect and A3CH showed a significant effect on FeLV, inhibiting the virus by a factor of 5. We also tested the simian lentivirus SIVagm-LucΔ*vif *and found that all cat A3s, except A3Cc, and dog A3H showed strong antiviral activity. Dog A3A did not reduce the infectivity of SIV (Figure 6e).

In summary, the feline A3Ca, A3Cb, and A3Cc proteins displayed very high activity only against FFVΔ*bet *while A3H and A3CH reduced FFVΔ*bet *infectivity much less. In contrast, only feline A3H and A3CH had a moderate inhibitory effect on Δ*vif *FIV, and A3CH weakly but significantly inhibited FeLV. The Vif protein of FIV counteracted feline A3H and A3CH, but failed to neutralize the antiviral activity of human and canine A3H. The FFV Bet protein mainly counteracted feline A3Ca, as recently shown [21], and A3Cb and A3Cc. We conclude that the various feline A3 proteins differentially target feline retroviruses with a remarkable virus-specific profile.

## Discussion

Phylogenetic analysis of the domestic cat APOBEC genes relative to human and dog demonstrated that cat and dog contain genes orthologous to human AICDA (AID), APOBEC1 (A1), A2, A3 and A4. The human A3 gene cluster on chromosome 22 spans 130 kb and contains seven genes that can be classified according to the presence/absence of the Z1a, Z1b and Z2 zinc-coordinating motifs [32,40]. Z1a, the A3C family, consists of human A3C, the carboxy- and amino-terminal domains of human A3DE and A3DF, and the amino-terminal domains of human A3B and A3G (Figure 4a). The Z1b group, the A3A family, contains human A3A and the carboxy-terminal domains of human A3B and A3G. The human A3H represents the Z2 zinc-finger domain. Accordingly, human A3B, A3G, A3DE, and A3F have two domains, while A3A, A3C, and A3H have one domain. Our analysis shows that the genome of the domestic cat contains three A3C genes (A3Ca to A3Cc) in addition to one A3H. The feline A3C genes have a single domain and are related to the human Z1a group but form their own cat specific lineage (Figure 4a). None of the domestic cat genes identified fall into the Z1b group. Cross-species BLAST analyses of the cat 1.9× genome sequence employing dog predicted genes for A3A and A3H using NCBIs cat WGS contig, trace and end-sequence databases failed to identify any cat gene other than A3C and A3H. Presumably, either the cat does not contain Z1b family genes or these genes are not represented in the 1.9× sequence. The fosmid DNA library and database described here provide an additional genomic DNA resource for isolation and characterization of feline genes involved in infectious and inherited disease. Since the fosmids in the library have been mapped to the 1.9× cat genomic sequence by end sequencing, it is not necessary to screen genomic libraries by hybridization or PCR to isolate genes of interest as with previous genomic libraries.

Human A3G and A3F have been shown to be active against HIV-1, which lacks the virion infectivity factor (*vif*) [13-16,41]. The Vif protein of HIV-1 exclusively binds and inactivates human A3 proteins in a species-specific way [17]. The D128K mutation in the human A3G gene altered the Vif interaction [42-44] and H186R correlated with slow AIDS progression in African American populations [45]. In felids three types of exogenous retroviruses are known: within Orthoretroviridae, FIV, a lentivirus related to HIV-1, and the gammaretrovirus FeLV; and, within Spumavirinae, FFV. FIV infects both wild and domestic felid species [6]. Similar to the diversification of SIV in African monkeys and apes, species-specific strains of FIV have been described [46]. But unlike SIV, which is detectable only in African species, FIV is endemic in African, South American and Asian Felidae [6]. For FeLV the prevalence in wild species is not known and limited studies on FFV supported the presence of FFV-related isolates in two species of the leopard cat lineage [47].

The ability of Vif proteins to counteract the antiviral activity of A3 proteins is specific for a virus-host system. Thus, while the HIV-Vif protein counteracts the human A3F and A3G proteins, it is not effective against cat A3H and A3CH, as we recently reported [24]. In contrast, FIV-Vif neutralizes the cat A3H and A3CH induced cytidine deamination. Since we could not detect homologous genes to A3F or A3G in the domestic cat genome, the essential role of controlling retrovirus replication seems to be covered by different A3 proteins in permissive mammals (humans and cats). Interestingly, neither human A3C nor A3H proteins are inhibitors of wild-type or Δ*vif *HIV-1 [18,32], supporting a host-specific genetic adaptation of A3 genes.

The *vif *gene of FIV is a relevant modulator of spreading virus infection, since FIV in which the *vif *gene was deleted showed a replication block in feline CrFK cells that express A3, as we showed [21,24,48]. Furthermore, in domestic cats experimentally infected with FIVpco isolated from *P. concolor*, the virus was controlled and the cats did not develop clinical signs associated with FIV infection. The restriction of FIVpco was attributed to feline A3 proteins, because the viral genomes of FIVpco grown in cats accumulated extensive G-to-A mutations [20]. It is likely that insufficient molecular recognition and inactivation of heterologous feline A3 by the Vif protein of FIVpco caused this attenuated virus infection. It is interesting to emphasize here that the *Puma *genus is the closest relative of the *Felis *genus, having diverged approximately 6.7 million years ago [34]. The ability of the cat A3 proteins to limit FIVpco infection while not being able to limit FIV infection may thus reflect the fact that the FIV infecting *F. catus *has evolved the potential to escape A3-mediated restriction of its host since the divergence of both felide lineages. Cat A3H and A3CH also showed some inhibitory activity against FeLV. In contrast to Δ*vif *FIV, these active antiviral proteins showed only weak antiviral activity against Δ*bet *FFV. Based on these findings, we conclude that specific feline A3 proteins selectively recognize and inactivate only defined subgroups of feline retroviruses, while 'non-adapted', heterologous retroviruses (for example, Δ*vif *SIVagm) can be inactivated by all three types of feA3s with the remarkable exception of feA3Cc. These data also reflect the fact that even without expression of Vif or Bet proteins, retroviruses differ, for unknown reasons, in their vulnerability to cognate A3 proteins.

The analysis of the genomic sequences and cDNAs of the cat A3 loci allowed us to identify three key-features not present in the primate A3 system: first, one ancestral A3C gene underwent two successive duplication events in recent times - the first event generated the ancestor of the present A3Cc gene and a second gene, which later on underwent a second duplication giving rise to the ancestors of the present A3Ca and A3Cb genes; second, the A3H gene in domestic cats is under purifying selection; and third, the double-domain A3CH is generated by a read-through transcription and alternative splicing of three genes. In addition, we detected at least 15 single nucleotide polymorphisms (SNPs) yielding non-synonymous substitutions in A3C genes of 8 different cat breeds. In primates, the seven A3 genes (A3A to A3H) are present as single copies on chromosome 22. In the genome of the domestic cat, we found three copies of the A3C gene (A3Ca, A3Cb and A3Cc) in a head-to-tail orientation on chromosome B4. The feline A3C genes encode proteins that are different to each other at six to seven amino acid sites. Phylogenetic analyses indicate that this gene triplication likely occurred by two consecutive duplication steps: one ancestral A3C gene duplicated to the ancestor of A3Cc and a second gene, which later duplicated, giving rise to A3Ca and A3Cb genes. The presence of a homologous A3Cc gene in *P. concolor *closely related to the cat A3Cc gene suggests that at least the first duplication event occurred before the divergence of the *Puma *and *Felis *lineages, approximately 6.7 million years ago. The phylogenetic position of *P. tigris *A3C basal to the three cat A3C genes suggests also that the first duplication event occurred after the divergence of the *Panthera *and the *Felis *lineages, approximately 10.8 million years ago. It is generally believed that the evolution of new protein functions after gene duplication plays an important role in the evolution of the diversity of organisms and typically allows for an increased specialization or function gain of the daughter genes [49,50]. In light of the seven A3 genes in primates, it is tempting to speculate that cats, like primates, were under a specific evolutionary pressure to increase the diversity of the co-expressed A3 proteins that provided additional fitness. Other mammals, such as rodents and eventually dogs, were either not faced by these infectious agents or managed to counteract retroviruses and related retroid elements in a way not involving A3 proteins.

While primate A3 genes are under an adaptive (positive) selection [9,32,33], we detected significant positive selection only for the feline A3C genes. Feline A3H was found to have more residues under purifying selection than feline A3C. It thus appears that restriction against an apparently innocuous virus (FFV), mediated in cat by the A3C genes, is under high selective pressure whereas A3H, which is active against two serious cat pathogens, FIV and FeLV, does not evolve adaptively. While we consider it unlikely that FFV has a strong but currently unidentified pathogenic potential, it is possible that restriction against additional pathogens has shaped this evolutionary pattern. For instance, the cat A3H may protect against highly conserved, endogenous retroelements or may act by targeting highly conserved, invariant viral structures of FIV and FeLV, both features that would result in purifying selection. It could also be that cat A3H took over additional important functions distinct from pathogen defense, inducing purifying selection. Finally, the combination of a conserved A3H domain carrying specifically optimized effecter functions with a highly adaptive module allowing recognition of changing targets may explain that the two-domain feA3CH is much more active against FIV and FeLV than the corresponding single-domain molecules that are either inactive (cat A3C) or have intermediate activity (cat A3H). We postulate that the generation of the fused A3CH transcript is an evolutionary way to gain a greater variety of proteins from a limited number of functional exons.

In order to express the potent anti-retroviral restriction factor A3CH, the cat has modularly combined sequences from A3Ca and A3Cb genes and the constant A3H domain. This was likely achieved by read-through transcription. Read-through transcription, also called transcription-induced chimerism, a mechanism where adjacent genes produce a single, fused RNA transcript, is found in at least 2-5% of human genes [51,52]. A general feature of human transcriptional read-through is that intergenic sequences in these RNAs are processed via the standard eukaryotic splicing machinery that removes introns from RNA transcripts. Intergenic splicing is favored in closely located gene pairs [51,52], as true for the triplicated feline A3C genes. Currently, the regulation of read-through transcription is uncharacterized and both *cis*-acting sequences and *trans*-acting suppressors/regulators of the termination machinery could regulate it. Since the cat A3CH protein displayed a significantly stronger antiviral activity against FIV and FeLV compared to the single-domain cat A3C and A3H proteins, the read-through transcription for cat A3 appears to be functionally relevant.

In this study we did not investigate whether the upstream genes (A3Ca and A3Cb) have legitimate transcription termination sites, and whether the downstream gene (A3H) has a legitimate promoter region. But consensus sequences for both regulatory elements are detectable using standard analysis tools (Figure S3 in Additional data file 3). This analysis showed considerable sequence similarity in the proximal 5' flanking sequences of all A3 genes except A3Cc, which has a unique upstream sequence, supporting the experimental data that A3Cc may have a unique transcription profile. In humans, A3 genes are differentially expressed in tissues associated with either endogenous or exogenous retroviral replication, including testes, the ovary and un-stimulated and stimulated peripheral blood lymphocytes (for a review, see [53]). Analysis of cDNA clones from domestic cat PBMCs, and MYA-1, CrFK and KE-R cells suggest that the cat A3 genes are also differentially expressed. An alternative possibility, however, is that the fused transcript of A3CH results from *trans*-splicing between separate pre-mRNAs of A3Ca, A3Cb and A3H genes. The amount of trans-splicing in mammals is unknown and only few examples have been described so far [54,55].

**Figure 7:**
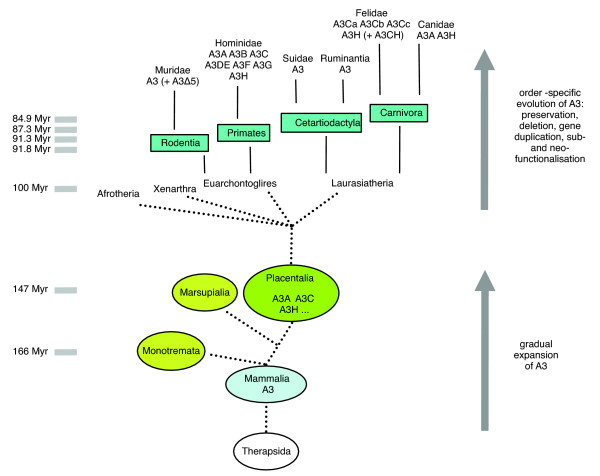
Model of mammalian A3 gene evolution. The model proposes the presence of several A3 genes in Placentalia before the separation of the super-orders Afrotheria, Xenarthra, Euarchontoglires and Laurasiatheria. According to the phylogenetic relationships among the extant A3 proteins, a host-specific evolution of the A3 genes during the early evolution of the Placentalia orders by means of preservation, deletion and/or gene duplication and concomitant subfunctionalization or neofunctionalization is inferred. Successive duplication events from a single ancestral A3 gene might have generated multiple A3 genes before basal radiation within Placentalia. The divergence times of taxons is in millions of years (Myr) ago. Basal radiation within Monotremata and Marsupialia is not shown. A3, APOBEC3; A3Δ5, A3 lacking exon 5 derived amino acids. Relationship of taxa and timing of mammalian evolution is based on [85], but please note that the timing is controversial [86]. The study of Wible *et al*. [87] supports a later diversification of the placentalia superorders following the Cretaceous-Tertiary (K/T) boundary 65 million years ago.

In the human A3 locus there is evidence of gene expansion. It was speculated that duplications of single-domain genes formed the two-domain A3B or A3G, and, subsequently, duplication of A3B or A3G formed A3F [30]. Primates and rodents are both part of the placentalia super-order of Euarchontoglires (synonymous with Supraprimates). While primates have seven A3 genes (A3A to A3H), mice and rats carry only a single A3 gene. In mice, in addition, a splice variant lacking exon 5 (A3Δ5) is expressed [17,56]. Based on these data, it was proposed that primates show a relatively recent and possibly unprecedented gene expansion [10] or that the gene expansion happened at the beginning of primate evolution [40]. The lack of data from other mammals was partially filled by Jónsson *et al*. [57], who characterized A3 proteins, designated A3Fs, from certain cetartiodactyla (cow, pig, sheep). Rodent and cetartiodactyla A3 proteins consist of two cytosine deaminase domains, where the amino-terminal domain is similar to human A3C and the carboxy-terminal domain shows the highest identity to human A3H [32]. This CH domain configuration is also found in feline A3CH described here. In our study we detected A3C and A3H genes in Felidae, while in the dog genome only genes corresponding to A3A and A3H are present. Cetartiodactyla and Carnivora are both grouped into the placentalia super-order of Laurasiatheria. Based on the presence of at least three different A3 types in Laurasiatheria, we propose that a certain set of A3 genes (A3A, A3C, A3H, or more) was already established before the separation of the placentalia order. During following evolution, this set of A3 genes was either preserved, fused, deleted or re-expanded depending on the specific requirements of the host-virus interactions (Figure 7). Following this idea, an initial expansion of a single A3 gene happened early in mammalian evolution, eventually before the appearance of the placentalia. Further studies on the absence or presence of A3 genes in the other placentalia super-orders (Xenathra and Afrotheria) and genomics in monotremata and marsupialia are necessary to critically evaluate our model of a two-stage evolution of A3 genes.

## Conclusion

Recent studies on the evolution of the primate APOBEC3 genes revealed a primate-specific gene amplification. We analyzed the genomic APOBEC3 region of the domestic cat (*F. catus*) and found a chromosomal APOBEC3 locus different to that of primates, rodents and dogs. Besides our detection of three very similar APOBEC3C genes and one APOBEC3H gene, the cat uses the mechanism of transcriptional read-through alternative splicing to generate a fifth antiviral APOBEC3 protein. The evolution of antiviral cytidine deaminases shows a strong placentalia family specific pattern. Our results indicate that three APOBEC3 genes (A, C and H) were present in the evolution of mammals before the placentalia super-orders separated.

## Materials and methods

### Fosmid database construction and utilization

A fosmid (pCC1) DNA library consisting of 693,504 clones containing an average insert size of approximately 40 kb of domestic cat (breed Abyssinian) genomic DNA arrayed in 1,806 384-well plates frozen at -80°C that was end-sequenced as part of the Feline Genome Project was obtained from Agencourt Bioscience, Inc. (Beverly, MA, USA) The bar-coded plates were recorded into a relational database using Filemaker Pro (Filemaker Inc., Santa Clara, CA, USA) according to their rack number and rack position in a -80°C freezer along with individual trace identification number, trace name, and clone identification number (which includes the location of each individual fosmid within the plate).

### Fosmid DNA isolation, PCR, and nucleotide sequencing

The appropriate well of a 384-well plate was picked using a culture loop and streaked across LB agar plates containing 12.5 μg/ml chloramphenicol and incubated at 37°C overnight. Fosmid DNA was isolated using a standard alkaline lysis procedure. DNA was diluted 1/1,000 in H_2_O and assayed by PCR containing 1.0 μM of primer pairs for feline sequence tagged sites, 200 μM deoxynucleoside 5'-triphosphates (dATP, dTTP, dCTP, dGTP), 10 mM Tris-HCl (pH 8.3), 50 mM KCl, 1.5 mM MgCl_2_, and 1 unit TAQ-Gold polymerase in a 20 μl reaction at 95°C for 4 minutes followed by 30 cycles of 95°C for 30 s, 60°C for 30 s and 72°C for 30 s and 72°C for 7 minutes at the end of cycles. PCR products were analyzed on 2% agarose gels containing 0.5× Tris-Borate-EDTA buffer (TBE) and positive PCR products were treated with exonuclease/shrimp alkaline phosphatase and sequenced using BigDye Terminator chemistry with the appropriate forward and reverse primer and analyzed using an ABI 3730XL as described previously [58]. Fosmids were further analyzed by end sequencing and/or transposon insertions. Sequences were assembled using Phred, Phrap, and Consed programs [59-61]. Genomic nucleotide sequences were analyzed using RepeatMasker [62] to identify repetitive elements. Genscan [63], Genewise [26] and Spidey [64] were used to identify coding sequences and Pipmaker [65] to visualize sequence features. Potential regulatory sites, including transcription factor binding sites, splice donor/acceptor sites and polyadenylation sites, were identified using Match™ [66], WebLogo [67,68] and MEME [27,28].

### SNP analysis of A3C genes in cat breeds

The following locus specific PCR primers were used for PCR reactions: A3Ca_exon2_F (GCTGTTCTTTGGGGATAGAGAG); A3Cb_exon2_F (GGTTGGGGGTAGGGCGGGCT); A3Cc_exon2_F (CACCCACCAGGGACAACTCG); A3Ca_exon2_R (TGGTTCTCTCCTGGAAACAGA); A3Cb_exon2_R (AGGCTGTGGCTGGGAGCAGA); A3Cc_exon2_R (TCTGAGAGATCAGAGGGCCG); A3Ca_exon3_4_F (CTCAAAAAAAAAGACAGGGCAGA); A3Cb_exon3_4_F (TCAAAAAAAAAGACAGGGCAGG); A3Cc_exon3_4_F (GATGGATGGATGGATAGATGGAT); A3Ca_exon3_4_R (GCTGGGGAGGGAGGTGCGGA); A3Cb_exon3_4_R (GCTGGGGAGGGAGGTGCGGT); and A3Cc_exon3_4_R (GCTGGGGAGGGAGGTGCGGC). DNA samples for eight domestic cat breeds (Abysinnian, Birman, British Shorthair, Egyptian Mau, Japanese Bobtail, Norwegian Forest, Sphynx, and Turkish van) were amplified by PCR as described above except that we used 25 ng of DNA sample and the annealing temperature was increased to 64-70°C depending on primer pair and DNA sample. PCR products were analyzed as described above and assembled using Sequencher 4.7 (Genecodes, Ann Arbor, MI, USA).

### Phylogenetic analysis

Reference sequences for human APOBEC genes and predicted dog APOBEC genes were identified from Ensembl, Refseq and the NCBI annotation of the dog 7× genome sequence, respectively. Domestic cat APOBEC genes were identified using human APOBEC reference gene sequences and were used to screen for traces containing related sequences. Genscan predicted genes were edited and aligned using Seqed and Clustalx (ABCC, NCI-Frederick, Frederick, MD, USA). The sequences used in the phylogenetic trees were aligned using Clustalw [69]. Manual correction of gaps and trimming of the homologous cDNA regions were accomplished with Jalview [70]. For consensus tree construction with bootstrap values, Seqboot (1,000 samples bootstrapping), Dnadist (maximum likelihood distance), Neighbor (UPGMA, jumble 10, different seed values), and Consense from the PHYLIP package [71] were run. The lengths of the branches were calculated with PAML3.15 (59; Model 0, Nssites 2, molecular clock, RateAncestor 1). The ancestral sequences for the different nodes were taken from the PAML result. Alternatively, cDNA sequences were translated and aligned at the amino acid level using MUSCLE [72], manually edited, filtered with GBLOCS [73], and then back-translated conserving the codon structure. Since some APOBEC proteins present two concatenated domains whereas others consist of a single domain, two-domain proteins were split and the individual domains analyzed separately. Parsimony analysis was performed with PROTPARS and with DNAPARS, from the PHYLIP package, after 1,000 cycles bootstrap. Distance analysis was performed using PROTDIST and Dnadist using both neighbor-joining and UPGMA (unweighted pair group method with arithmetic mean), and combining the results. Bayesian phylogenetic inference was performed with BEAST v1.4.6 [74], partitioning the nucleotide sequence following the three codon positions, under the Hasegawa-Kishino-Yano model of evolution, using a strict clock and a Jeffreys prior distribution for the coalescent population size parameter, with no phylogenetic constraints, in two independent chains of 10,000,000 generations, sampling every 1,000 generations. Maximum likelihood analysis were performed with RAxML [75,76] under the Wheland and Goldman model of evolution, executing 500 non-parametric bootstaps.

### Ka/Ks analysis of sequence pairs

Ka/Ks values between sequence pairs out of the alignments were calculated using the Diverge program (Wisconsin Package, Accelrys Inc (San Diego, CA, USA). For the sliding window approach (window 300 bp, slide 50 bp) the program was run with the appropriate part of the sequences. Test for positive selection: the cat A3C and A3H trees were further tested for the presence of positive selection among amino acid sites using PAML. The likelihood ratio test was used to compare the evolutionary models M1 (neutral) and M2 (selection), M7 (beta) and M8 (beta&omega variations) [77,78]. The likelihood ratio test statistic was calculated by 2 × logΔ, where Δ = L0 (null modeldata)/L1 (alternative modeldata), L0 is the likelihood estimate for the simple model, and L1 is the likelihood estimate for the model with more free parameters. The degrees of freedom were determined by the difference in the number of free parameters between the null and alternative models, and the test statistic was approximated to a χ^2 ^distribution to determine statistical significance. In models M2 and M8, an empirical Bayesian approach is used to calculate the posterior probability that an amino acid site fits in each site class and sites with a high posterior probability of falling into the class of ω of >1 are considered to be under positive selection. Additionally, we identified the residues under positive and purifying selection using the webserver Selecton version 2.2 [37], submitting the alignments of the cat A3C and A3H sequences.

### Cells and transfections

Human cell line 293T, and feline cell lines CrFK (ATCC CCL-94, feline kidney cells) and KE-R (feline embryonic fibroblast cells, a gift of Roland Riebe, Friedrich-Loeffler Institut, Riems) were maintained in Dulbecco's high glucose modified Eagle's medium (Biochrom, Berlin, Germany; Dulbecco's modified Eagle's medium complete) supplemented with 10% heat-inactivated fetal bovine serum (FBS), 0.29 mg/ml L-glutamine, and 100 units/ml penicillin/streptomycin. Feline T-cell lines MYA-1 (ATCC CRL-2417) and FeT-1C (ATCC CRL-11968) were cultured in complete RPMI 1640, 0.29 mg/ml L-glutamine, 10 mM HEPES, and 1.0 mM sodium pyruvate and supplemented with 0.05 mM 2-mercaptoethanol, 100 units/ml human recombinant interleukin-2 and 10% heat-inactivated FBS, and 100 units/ml penicillin/streptomycin. Plasmid transfection into 293T cells was done with Lipofectamine 2000 according to the manufacturer's instructions (Invitrogen, Karlsruhe, Germany). PBMCs of Felidae and a dog were isolated from EDTA- or heparin-treated whole blood by Histopaque-1077 (Sigma, Taufkirchen, Germany) gradient centrifugation and cultured after activation with phytohemagglutinin (PHA; 3 μg/ml) for 3 days in RPMI medium 1640 containing 15% FBS, 5 × 10^-5 ^M 2-mercaptoethanol, 2 mM L-glutamine, and 100 units of human recombinant interleukin-2 per ml at 37°C and 5% CO_2_. Blood of one dog (*Canis familiaris*) of the breed Australian shepherd was obtained from Karin Kliemann. Blood of *F. catus *was obtained from two cats of unknown breed from the Max Planck Institute for Brain Research, Frankfurt, Germany. Blood from one each of lion (*P. leo bleyenberghi*), tigers (*P. tigris sumatrae *and *P. tigris corbetti*), leopard (*P. pardus japonensis*), lynx (*L. lynx*) and puma (*P. concolor*) were obtained from the Halle Zoo, Germany.

### Viruses and infections

FIV single-cycle luciferase vectors (FIV-Luc) were produced by cotransfecting 293T cells with: pFP93 (derived from clone FIV-34TF10, a gift of Eric M Poeschla [79]), which does not express *vif*; or pCPRΔEnv (derived from clone FIV-PPR, a gift of Garry P Nolan [80]), which does express *vif*; pLinSin; a VSV-G expression plasmid pMD.G [81]; and indicated APOBEC3-HA expression plasmids or empty vector (pcDNA3.1(+) (Invitrogen) or pcDNA3.1(+)zeo (Invitrogen). Vector pLiNSin was derived from pGiNSin, a self-inactivating (Sin) vector variant of pGiNWF [79], which is a minimal bi-cistronic FIV transfer vector plasmid coding for enhanced-green-fluorescent-protein (EGFP) and neomycin phosphotransferase containing Woodchuck hepatitis virus posttranscriptional regulatory element (WPRE) and FIV central DNA flap. The EGFP gene in pGiNSin was replaced by the firefly luciferase gene (luc3) using the restriction sites *Age*I and *Apa*I. The luciferase gene was amplified by overlapping PCR using pSIVagmLuc [17] as template and primers (feLuc3_1.f 5'-TCCACCGGTCGCCACCATGGAAGACGCCAA-3' (*Age*I-restriction site underlined); feLuc3_1.r 5'-CGTTGGCCGCTTTACACGGCGATC-3'; feLuc3_2.f 5'-GATCGCCGTGTAAAGCGGCCAACG-3'; feLuc3_2.r 5'-TTCCGGGCCCTCACATTGCCAAA-3' (*Apa*I-restriction site underlined)), subcloned in pCR4Blunt-TOPO (Invitrogen), and sequence verified. FeLV reporter virions were produced by transfection of 293T cells with FeLV-A clone p61E-FeLV [82], MLV-EGFP transfer vector pMgEGFP-ΔLNGFR [83], VSV-G expression plasmid and the indicated expression plasmid for APOBEC3-HA or pcDNA3.1(+). Reverse-transcription of viruses was determined by Cavidi HS kit Lenti RT or C-type RT (Cavidi Tech, Uppsala, Sweden). For reporter virus infections, HOS cells were seeded at 2.0 × 10^3 ^cells/well a day before transduction in 96-well plates and then infected with reporter virus stocks normalized for RT. Firefly luciferase activity was measured three days later with a Steadylite HTS reporter gene assay system (PerkinElmer, Cologne, Germany) according to the manufacturer's directions on a Berthold MicroLumat Plus luminometer. Expression of EGFP was analyzed by flow cytometry. Propagation of wild-type and Bet-deficient FFV (pCF-7 and pCF-Bet-BBtr [21]), contransfection with defined APOBEC3-HA expression plasmids, titration of FFV infectivity, and detection of FFV proteins was done as described previously [21].

### Sequencing of viral reverse transcripts

293T cells (1 × 10^6^) were infected with DNase I (Roche, Mannheim, Germany) treated wild-type or Δ*vif *FIV(VSV-G) (1,000 pg RT) using vector pGiNSin produced in 293T cells together with feline APOBEC3s or pcDNA3.1(+). At 10 h post-infection, cells were washed with phosphate-buffered saline and DNA was isolated using DNeasy DNA isolation kit (Qiagen, Hilden, Germany). A 300 bp fragment of the *egfp *gene was amplified using *Taq *DNA polymerase and the primers eGFP.fw (5'-cgtccaggagcgcaccatcttctt-3') and eGFP.rv (5'-atcgcgcttctcgttggggtcttt-3'). Each of 30 cycles was run at 94° for 30 s, 58° for 1 minute, and 72° for 2 minutes, and PCR products were cloned into TOPO TA-cloning pCR4 vector (Invitrogen) and sequenced. The nucleotide sequences of at least eight independent clones were analyzed.

### Plasmids

All APOBEC3s are expressed as carboxy-terminal HA-tagged proteins (APOBEC3-HA). Feline APOBEC3Ca (previously termed feAPOBEC3, feA3, GenBank accession no. AY971954) was described in [21]. Feline APOBEC3Cb and APOBEC3Cc were similarly constructed. Feline APOBEC3H and feline APOBEC3CH cDNAs were identified by using 5' and 3' RACE reactions (5'/3'-RACE kit, Roche Diagnostics) employing total RNA from CrFK cells [24]. APOBEC3 cDNAs of big cats were amplified from cDNA of PBMCs after activation with PHA (3 μg/ml) and *Pwo *polymerase (Roche Diagnostics) was applied. The primers were: for A3C forward primer fApo3F-18 (5'-TAGAAGCTTACCAAGGCTGGCGAGAGGAATGG-3', HindIII site underlined) and reverse primer fAPO3F-19 (5'-AGCTCGAGTCAAGCGTAATCTGGAACATCGTATGGATACCTAAGGATTTCTTGAAGCTCTGC-3' (*Xho*I site underlined)); for A3H, forward primer fAPO-29 (5'-TGCATCGGTACCTGGAGGCAGCCTGGGAGGTG-3' (*Kpn*I site underlined)) and reverse primer fAPO-28 (5'-AGCTCGAGTCAAGCGTAATCTGGAACATCGTATGGATATTCAAGTTTCAAATTTCTGAAG-3' (*Xho*I site underlined)). Thirty cycles were run at 94°C for 30 s, 58°C for 1 minute, and 72°C for 2 minutes. PCR products were cloned into pcDNA3.1(+) using restriction sites *Hin*dIII and *Xho*I (for A3C) or *Kpn*I and *Xho*I (for A3H) and sequenced. Expression plasmids of 3'-HA-tagged dog (*C. familiaris*) APOBEC3A (GenBank accession number. XM_847690.1) and APOBEC3H (GenBank accession number XM_538369.2) were generated by PCR. For dog A3A the template was expressed sequence tag clone nas31ho7 5' (GenBank accession number DN874273), obtained through Graeme Wistow from the National Eye Institute, Bethesda, USA, and *Pwo *polymerase was used. The forward primer was dog-APO-11 (5'-TGCAGGTACCCCGCGGACATGGAGGCTGGCC-3' (*Kpn*I site underlined)), and the reverse primer was Dog-APO-10 (5' AGTGCGGCCGCTCAAGCGTAATCTGGAACATCGTATGGATATAGGCAGACTGAGGTCCATCC-3' (*Not*I site underlined)). Dog A3H cDNA was amplified from cDNA of PBMCs after activation with PHA (3 μg/ml), using forward primer Dog-APO-7 (5'-TGCAGGTACCCCACGATGAATCCACTACAAGAAGA-3' (*Kpn*I site underlined)), and reverse primer Dog-APO-8 (5'-AGTGCGGCCGCTCAAGCGTAATCTGGAACATCGTATGGATAAAGTCTCAAATTTCTGAAGTC-3' (*Not*I site underlined)), and *Pwo *polymerase (Roche). Thirty cycles were run at 94°C for 30 s, 58°C for 1 minute, and 72°C for 2 minutes. PCR products were digested by *Kpn*I and *Not*I, introduced into pcDNA3.1(+)-Zeo (Invitrogen) and correct clones identified by sequencing. For generation of the pGex-feAPOBEC3CH-linker the feline A3CH linker region containing the sequence from amino acids 190-244 of the feline A3CH was cloned into a pGEX-4T3 Vector (Amersham Bioscience, Freiburg, Germany) by PCR (forward primer, 5'-CGAGTCGAATTCCCTTAGTCCCGGCCAACAAAGAAAAAGAGAC-3'; reverse primer, 5'-GCATGAGTCGACTGTGGGTCTGGGCAAGAGGAAGG-3'; the introduced restriction sites for *Eco*RI and *Sal*I are underlined). Purified DNA fragments were fused in frame between the 5' GST domain and the 3' SV40 tag (KPPTPPPEPET) of correspondingly digested pGEX4T3tag derivatives [84]. Clones were identified by restriction enzyme digestion and DNA sequencing. pcFIV.Vif-V5 is an expression plasmid for the codon-optimized *vif *of FIV-34TF10 (GenBank accession number M25381). It was generated by cloning the codon-optimized *vif *gene, 3' fused to the V5-tag, made by Geneart (Regensburg, Germany) into pcDNA3.1(+) using the *Kpn*I and *Not*I restriction sites; a 3'-WPRE element was included in the *Not*I and *Apa*I sites.

### Expression studies

Expression studies of feline APOBEC3C RNAs in CrFK, KE-R, MYA-1, and Fet1C cells and PHA-activated PMBCs of cat and other Felidae were done by RT-PCR using 2 μg of total RNA (RNeasy mini kit, Qiagen) and cDNA made by SuperScript III RT (Invitrogen). For feline APOBEC3Cs the primers were forward fAPO3F-9 (5'-TGGAGGCAGCCTGGGAGGTG-3'), and reverse fAPO3F-15 (5'-GCGAGACGCAAGGAACAGCAG-3'). For feline APOBEC3H the primers were forward fAPO3F-9 and reverse fAPO-26 (5'-CTGCCCGAAGGCACCCTAATTC-3'), or, alternatively, forward feA3H.fw (5'-ATGAATCCACTACAGGAAGTCATAT-3') and reverse feA3H.rv (5-TCATTCAAGTTTCAAATTTCTGAAG-3'). For feline APOBEC3CH the primers were forward feAPO3CH.fw (5'-ATGGAGCCCTGGCGCCCCAGCCCAA-3') and reverse fAPO-27 (5'-TCGTACTCGAGGCAGTTTATGAAGCATTGAGATGC-3'). *Pwo *polymerase (Roche) was used for cloning and *Taq *polymerase (Fermentas, St. Leon-Rot, Germany) for diagnostic PCR. PCRs were run for 30 cycles at 94°C for 30 s, annealing for 1 minute at 60°C for feA3C, 58°C for feA3H, and 59°C for feA3CH, and 72°C for 2 minutes. PCR products were cloned in TOPO-vectors (Invitrogen) and sequenced.

### Immunoblot analysis

Cells were cotransfected with plasmids for FFV, FIV, FeLV and APOBEC3-HA expression plasmids and lysates were prepared two days later. Cell lysates were prepared by removing the medium from transfected cells, washing them with phosphate-buffered saline, and lysing them in lysis buffer. Protein in the lysates was quantified using Coomassie blue reagent (Bio-Rad, Munich, Germany). Lysates containing 20 μg of protein were separated by SDS-PAGE and transferred to polyvinylidene difluoride filters or nitrocellulose membranes. Filters were probed with anti-HA antibody (1:6,000 dilution, MMS-101P; Covance, Münster, Germany), or mouse anti-α-tubulin (1:4,000 dilution, clone B5-1-2, Sigma-Aldrich) or polyclonal rabbit antibody against the linker region of feline A3CH (1:250 dilution) followed by horseradish peroxidase-conjugated rabbit anti-mouse antibody (α-mouse-IgG-HRP, Amersham Biosciences) or Protein A-peroxidase (Sigma) and developed with ECL chemiluminescence reagents (Amersham Biosciences). A polyclonal mono-specific rabbit antiserum against the feline A3CH linker sequence (A3CH amino acid residues 190-244) was generated using a GST-fusion protein made with a pGex-feAPOBEC3CH-linker. The GST-fusion protein was purified as described and used for vaccination of rabbits [21].

### Data deposition

The sequences reported here have been deposited in the GenBank database: feline A3 genomic locus, including feA3Ca, feA3Cb, feA3Cc, and feA3H (EU109281); feA3Ca (AY971954); feA3C isolate X (EU057980); feA3C isolate Y (EU057981); feA3H (EU011792); feA3H (Ex2 5'Δ) (EF173020); feA3CH (EF173021); feA1, feA2, feA4, and AICDA (sequences derived from cat genomic project AANG00000000.1); leopard A3C (DQ205650); tiger A3C#1 (DQ093375); tiger A3C#2 (EU016361); tiger A3C#3 (EU016362); lion A3C#1 (EU007543); lion A3C#2 (EU007544); lynx A3C#1 (EU007546); lynx A3C#2 (EU016363); lynx A3C#5 (EU007547); lynx A3C#6 (EU007548); puma A3C (EU007545); leopard A3H (EU007551); tiger A3H (EU007550); lion A3H (EU007549); lynx A3H (EU007553); puma A3H (EU007552); codon optimized *vif *of FIV (EF989123).

## Abbreviations

A3, APOBEC3; AICDA, activation-induced cytidine deaminase (also known as AID; APOBEC3, apolipoprotein B mRNA-editing catalytic polypeptide 3; FBS, fetal bovine serum; FeLV, feline leukemia virus; FFV, feline foamy virus; FIV, feline immunodeficiency virus; HA, hemagglutinin; HIV, human immunodeficiency virus; Luc, luciferase; NCBI, National Center for Biotechnology Information; PAML, Phylogenetic Analysis by Maximum Likelihood; PBMCs, peripheral blood mononuclear cells; PHA, phytohemagglutinin; RACE, rapid amplification of cDNA ends; RT-PCR, reverse-transcriptase PCR; SIV, simian immunodeficiency virus; SNP, single nucleotide polymorphism; Vif, viral infectivity factor; VSV-G, vesicular stomatitis virus-G protein; WGS, whole genome shotgun.

## Authors' contributions

CM, ML and NY designed the study, analyzed data and wrote the manuscript. TB characterized the genomic A3 locus, characterized the SNPs and wrote the manuscript. AHW and IGB performed bioinformatics and wrote the manuscript. JZ, SC and MB performed cell culture and biochemical experiments. KC and SOB contributed material/reagents/analysis tools and analyzed data. JT characterized and provided reagents.

## Additional data files

The following additional data are available with the online version of this paper. Additional data file 1 provides supplemental information about the calculation of Ka/Ks. Additional file 2 provides tables showing percent identity of cat A3C introns (supplementary Table 1), Ka/Ks ratios of cat A3s (supplementary Tables 2 and 3), A3C SNPs of cat breeds (supplementary Table 4), percent identities of all described A3C and A3H cDNAs and proteins (supplementary Tables 5 and 6) and results of different evolutionary models (supplementary Table 7). Additional file 3 provides supplementary figures. Supplementary Figure 1 shows amino acid alignments of feline APOBEC3 proteins. Supplementary Figure 2 shows amino acid alignments of feline, canine and human APOBEC3 proteins. Supplementary Figure 3 contains sequences and positions of predicted transcription factor binding sites. Supplementary Figure 4 shows analysis of evolutionary selection by the sliding window approach. Supplementary Figure 5 presents analysis of cytidine deamination of FIV by feline A3s.

## Supplementary Material

Additional data file 1 Information about the calculation of Ka/Ks. Click here for file

Additional data file 2 Supplementary Table 1 lists percent identity of cat A3C introns. Supplementary Tables 2 and 3 list Ka/Ks ratios of cat A3s. Supplementary Table 4 lists A3C SNPs of cat breeds. Supplementary Tables 5 and 6 list percent identities of all described A3C and A3H cDNAs and proteins. Supplementary Table 7 lists results of different evolutionary models. Click here for file

Additional data file 3 Figure S1: comparison of amino acid sequences of the feline A3C genes. Predicted amino acid sequence of the feline APOBEC3Ca, APOBEC3Cb and APOBEC3Cc proteins in comparison with the two additional variant cDNAs detected (A3Cx and A3Cy) in cat PBMCs. The zinc coordination domain is indicated. Residues different to A3Ca are shown in bold. Figure S2: amino acid alignment of feline, canine and human APOBEC3 proteins. **(a) **Amino acid alignment of feline APOBEC3Ca, APOBEC3Cb, APOBEC3Cc, human APOBEC3C, APOBEC3F and murine APOBEC3 NT. **(b) **Amino acid alignment of feline, canine, human APOBEC3H and murine APOBEC3 NT. **(c) **Amino acid alignment of human, canine APOBEC3A and human APOBEC3G CT. The zinc-coordinating domains are indicated. CT, carboxyl-terminal domain; NT, amino-terminal domain. Figure S3: prediction of transcription factor binding sites. Potential transcription factor binding sites in A3 cluster of the domestic cat in the region 1.1 kb upstream, including 100 bp of the predicted exon 1 for each gene (A3Ca, A3Cb, A3Cc and A3H) using ClustalW. The individual 5' flanking sequences were analyzed using the program Match, which uses a library of nucleotide weight matrices from the TRANSFAC6.0 database for transcription factor binding sites. Figure S4: analysis of Ka/Ks. Sliding window (300 bp window, 50 bp slide) analysis of Ka and Ks was performed on pairs of **(a) **cat A3C sequences and **(b) **cat A3H sequences and compared with corresponding selected felid and human sequences. Ka/Ks is plotted against the length of the coding region of the mRNAs with a schematic presentation of protein domains along the x-axis. Figure S5: analysis of cytidine deamination in the genomes of FIV by feline APOBEC3s. **(a) **A fragment of the reporter gene (*egfp*) was amplified from reverse transcripts of Δ*vif *FIV (left panel) or wild-type FIV (right panel) generated in the presence of the indicated feline APOBEC3s 10 h post-infection. A total of eight independent nucleotide sequences were determined. The mutations in the clones of each group are shown. Each mutation is indicated and coded with respect to nucleotide mutation. **(b) **The number of G→A changes per 100 Gs is shown. **(c) **Comparison of the dinucleotide sequence context of G→A mutations in the positive-strand DNA of Δ*vif *FIV derived from feA3-expressing 293T cells. **(d) **Sequence characteristics of Δ*vif *FIV DNA genomes of virions derived from 293T-expressing feline APOBEC3 proteins or empty expression plasmid (vector). Click here for file
