# Supplementary material for: Functions, structure, and read-through alternative splicing of feline APOBEC3 genes
Source: Genome Biol. 2008 Mar 3;9(3):R48. doi: 10.1186/gb-2008-9-3-r48 (PMC2397500; doi:10.1186/gb-2008-9-3-r48)
Supplement: Additional data file 1 — Information about the calculation of Ka/Ks. [file gb-2008-9-3-r48-S1.doc]

**Supplemental Information**

**Supplemental information to the calculation of Ka/Ks:**

The A3C and A3H genes were further tested for the presence of positive selection among amino acid sites using the Phylogenetic Analysis by Maximum Likelihood (PAML) software program version 3.15 [35, 36]. The likelihood ratio test (LRT) was used to compare the models M1 (neutral) and M2 (selection), M7 (beta) and M8 (beta&omega variations) [77, 78]. Comparing model M1 to model M2 and model M7 to model M8 tests the hypothesis that a gene contains amino acid sites that are under positive selection. While models M1 and M2 use a discrete number of site classes for ω values (allowing for two and three distinct ω values, respectively), models M7 and M8 use a discrete approximation of a continuous distribution of ω values (allowing for 10 and 11 distinct ω values, respectively). Models M2 and M8 identify codons that fall into a category where ω is >1 and therefore may be under positive selection. In models M2 and M8, an empirical Bayesian approach is used to calculate the posterior probability that an amino acid site fits in each site class and sites with a high posterior probability of falling into the class of ω of >1 are considered to be under positive selection. The two comparisons yielded in both cases (A3C and A3H) better estimates for the models assuming positive selection, M2 and M8, respectively (table S4). For M1-M2 comparison, A3H revealed 2 delta = 1.32, P < 0.4 with df = 2, and A3C revealed 2 delta = 30.3, P < 0.0001 with df = 2. For M7-M8 comparisons, A3H revealed the same result as for the M1-M2 comparison, A3C revealed 2 delta = 31.43, P < 0.0001 with df = 2. In summary, the significance was much higher in the case of A3C. M2 and M8 models produced largely consistent results regarding sites, which are probably under positive selection. Both models indicated the same sites for A3H and A3C to be likely under diversifying selection (table S4). On the other hand, the data of this analysis are in favor that vast majority of the sites in A3H and many sites in A3C are under purifying selection.
