# Supplementary material for: Functions, structure, and read-through alternative splicing of feline APOBEC3 genes
Source: Genome Biol. 2008 Mar 3;9(3):R48. doi: 10.1186/gb-2008-9-3-r48 (PMC2397500; doi:10.1186/gb-2008-9-3-r48)
Supplement: Additional data file 2 — Supplementary Table 1 lists percent identity of cat A3C introns. Supplementary Tables 2 and 3 list Ka/Ks ratios of cat A3s. Supplementary Table 4 lists A3C SNPs of cat breeds. Supplementary Tables 5 and 6 list percent identities of all described A3C and A3H cDNAs and proteins. Supplementary Table 7 lists results of different evolutionary models. [file gb-2008-9-3-r48-S2.doc]

**Supplemental Tables**

**Supplemental Table 1. Percent identity of cat A3C introns**

|  |  |  | **Percent Identity Introns feA3Cs** | |
| --- | --- | --- | --- | --- |
|  |  |  |  |  |
|  |  | **Size (bp)** | **A3Ca** | **A3Cb** |
| **Intron 1** | **A3Ca** | 3220 |  |  |
|  | **A3Cb** | 3216 | 99.192 (3216, 3) |  |
|  | **A3Cc** | 1268 | 68.215 (1268, 9) | 67.225 (1268, 7) |
|  |  |  |  |  |
|  |  |  |  |  |
| **Intron 2** | **A3Ca** | 2429 |  |  |
|  | **A3Cb** | 2474 | 98.721 (2429, 6) |  |
|  | **A3Cc** | 1613 | 85.581 (1613, 8) | 84.864 (1613, 6) |
|  |  |  |  |  |
|  |  |  |  |  |
| **Intron 3** | **A3Ca** | 230 |  |  |
|  | **A3Cb** | 230 | 98.696 (230, 0) |  |
|  | **A3Cc** | 234 | 95.217 (230, 1) | 96.087 (230, 1) |

(length, gaps)

**Supplemental Table 2. Ka/Ks of cat A3Ca versus other felid A3C cDNA sequences**

|  | Cat A3Ca | Cat A3Cb | Cat A3Cc | Puma A3C | Lion A3C#2 | Lion A3C#1 | Leopard A3C | Tiger A3C#1 | Tiger A3C#2 | Tiger A3C#3 | Lynx A3C#1 | Lynx A3C#2 | Lynx A3C#5 |
| --- | --- | --- | --- | --- | --- | --- | --- | --- | --- | --- | --- | --- | --- |
| Cat A3Cb | 3.222 |  |  |  |  |  |  |  |  |  |  |  |  |
| Cat A3Cc | 0.658 | 1.504 |  |  |  |  |  |  |  |  |  |  |  |
| Puma A3C | 1.213 | 1.464 | 0.949 |  |  |  |  |  |  |  |  |  |  |
| Lion A3C#2 | 0.951 | 1.227 | 1.402 | 1.125 |  |  |  |  |  |  |  |  |  |
| Lion A3C#1 | 1.54 | 2.018 | 1.765 | 1.228 | 2.504 |  |  |  |  |  |  |  |  |
| Leopard A3C | 1.56 | 2.044 | 1.807 | 1.388 | 2.538 | infinity |  |  |  |  |  |  |  |
| Tiger A3C#1 | 0.644 | 0.73 | 0.81 | 0.831 | 2.708 | 2.223 | 1.983 |  |  |  |  |  |  |
| Tiger A3C#2 | 0.797 | 0.903 | 0.976 | 1.052 | 4.16 | 2.727 | 2.478 | infinity |  |  |  |  |  |
| Tiger A3C#3 | 0.591 | 0.79 | 0.755 | 0.756 | 2.185 | 2.043 | 1.806 | infinity | infinity |  |  |  |  |
| Lynx A3C#1 | 0.911 | 0.949 | 1.434 | 1.372 | 1.431 | 2.534 | 2.536 | 1.163 | 1.349 | 1.096 |  |  |  |
| Lynx A3C#2 | 0.854 | 0.887 | 1.256 | 1.354 | 0.895 | 0.972 | 0.984 | 0.724 | 0.833 | 0.689 | 0.531 |  |  |
| Lynx A3C#5 | 0.897 | 0.925 | 1.239 | 0.998 | 0.678 | 0.803 | 0.863 | 0.581 | 0.68 | 0.549 | 0.599 | 2.299 |  |
| Lynx A3C#6 | 1.661 | 1.565 | 2.458 | 2.842 | 1.062 | 1.736 | 1.757 | 0.97 | 1.127 | 0.917 | 0.461 | 0.763 | 0.827 |

**Supplemental Table 3. Ka/Ks of cat A3H versus other felid A3H cDNA sequences**

|  | Cat A3H | Leopard A3H | Lion A3H | Puma A3H | Tiger A3H |
| --- | --- | --- | --- | --- | --- |
| Leopard A3H | 0.829 |  |  |  |  |
| Lion A3H | 0.83 | infinity |  |  |  |
| Puma A3H | 0.794 | 1.564 | 1.564 |  |  |
| Tiger A3H | 0.747 | infinity | infinity | 1.423 |  |
| Lynx A3H | 0.886 | 3.383 | 3.383 | 1.755 | 2.964 |

Note: infinity means Ks=0; Ka/Ks could not be calculated.

**Supplemental Table 4. Single nucleotide polymorphisms in APOBEC3C genes in eight domestic cat breeds**

| **Breed** | **Sample Number** | **Gene** | **Exon** | **Nucleotide^a^** | **Amino Acid** |
| --- | --- | --- | --- | --- | --- |
| Abysinnian | Fca6162 | A3Cb | 3 | C324T | Silent^b^ |
| Birman | Fca611 | A3Cb | 2 | C35T | T12M |
| Birman | Fca611 | A3Cb | 4 | G536C | G179A^b^ |
| Birman | Fca611 | A3Cb | 4 | T545C | F182S^b^ |
| Birman | Fca611 | A3Cb | 4 | A556G | K186E^b^ |
| British Shorthair | Fca2588 | A3Cb | 2 | C35T | T12M |
| British Shorthair | Fca2588 | A3Cb | 3 | C324T | Silent |
| British Shorthair | Fca2588 | A3Cb | 3 | C393A | D131E |
| British Shorthair | Fca2588 | A3Cb | 4 | G515A | R172Q |
| British Shorthair | Fca2588 | A3Cb | 4 | G536A | G179D |
| British Shorthair | Fca2588 | A3Cb | 4 | C546A | F182L |
| British Shorthair | Fca2588 | A3Cb | 4 | A556G | K186E |
| Egyptian Mau | Fca137 | A3Cb | 2 | C35T | T12M^b^ |
| Egyptian Mau | Fca137 | A3Cb | 2 | C162G | D54E^b^ |
| Egyptian Mau | Fca137 | A3Cb | 3 | G231A | Stop^b^ |
| Egyptian Mau | Fca137 | A3Cb | 3 | C324T | Silent^b^ |
| Egyptian Mau | Fca137 | A3Cb | 3 | C393A | D131E^b^ |
| Egyptian Mau | Fca137 | A3Cb | 4 | A469G | K157E^b^ |
| Egyptian Mau | Fca137 | A3Cb | 4 | C472T | H158Y^b^ |
| Egyptian Mau | Fca137 | A3Cb | 4 | G493T | D165Y^b^ |
| Egyptian Mau | Fca137 | A3Cb | 4 | C496A | H166N^b^ |
| Egyptian Mau | Fca137 | A3Cb | 4 | G515A | R172Q^b^ |
| Egyptian Mau | Fca137 | A3Cb | 4 | G536A | G179D^b^ |
| Egyptian Mau | Fca137 | A3Cb | 4 | A556G | K186E^b^ |
| Japanese Bobtail | Fca2673 | A3Cb | 2 | C35T | T12M^b^ |
| Japanese Bobtail | Fca2673 | A3Cb | 3 | del261 | Null^b^ |
| Japanese Bobtail | Fca2673 | A3Cb | 4 | G536A | G179D^b^ |
| Japanese Bobtail | Fca2673 | A3Cb | 4 | A556G | K186E^b^ |
| Sphynx | Fca1497 | A3Cb | 2 | C35T | T12M^b^ |
| Sphynx | Fca1497 | A3Cb | 3 | C324T | Silent^b^ |
| Sphynx | Fca1497 | A3Cb | 4 | A469G | K157E^b^ |
| Sphynx | Fca1497 | A3Cb | 4 | C472T | H158Y^b^ |
| Sphynx | Fca1497 | A3Cb | 4 | G493T | D165Y^b^ |
| Sphynx | Fca1497 | A3Cb | 4 | C496A | H166N^b^ |
| Sphynx | Fca1497 | A3Cb | 4 | G515A | R172Q^b^ |
| Sphynx | Fca1497 | A3Cb | 4 | G536A | G179D^b^ |
| Sphynx | Fca1497 | A3Cb | 4 | C546A | F182L^b^ |
| Sphynx | Fca1497 | A3Cb | 4 | A556G | K186E^b^ |
| Norwegian Forest | Fca2156 | A3Cb | 2 | C35T | T12M |
| Norwegian Forest | Fca2156 | A3Cb | 3 | C324T | Silent |
| Norwegian Forest | Fca2156 | A3Cb | 3 | C393A | D131E |
| Norwegian Forest | Fca2156 | A3Cb | 4 | G515A | R172Q |
| Norwegian Forest | Fca2156 | A3Cb | 4 | G536A | G179D |
| Norwegian Forest | Fca2156 | A3Cb | 4 | C546A | F182L |
| Norwegian Forest | Fca2156 | A3Cb | 4 | A556G | K186E |
| Turkish van | Fca2417 | A3Cb | 2 | C35T | T12M^b^ |
| Turkish van | Fca2417 | A3Cb | 2 | C162G | D54E^b^ |
| Turkish van | Fca2417 | A3Cb | 3 | C324T | Silent^b^ |
| Turkish van | Fca2417 | A3Cb | 4 | A469G | K157E^b^ |
| Turkish van | Fca2417 | A3Cb | 4 | C472T | H158Y^b^ |
| Turkish van | Fca2417 | A3Cb | 4 | G493T | D165Y^b^ |
| Turkish van | Fca2417 | A3Cb | 4 | C496A | H166N^b^ |
| Turkish van | Fca2417 | A3Cb | 4 | G536A | G179D^b^ |
| Turkish van | Fca2417 | A3Cb | 4 | A556G | K186E^b^ |
| Abysinnian | Fca6162 | A3Cc | 2 | G63T | Silent |
| Birman | Fca611 | A3Cc | 3 | T293A | W65R |
| Birman | Fca611 | A3Cc | 3 | C387G | F129L |
| Birman | Fca611 | A3Cc | 3 | A389G | Y130W |
| Birman | Fca611 | A3Cc | 3 | C390G | Y130W |
| Birman | Fca611 | A3Cc | 3 | C391G | H131D |
| Birman | Fca611 | A3Cc | 4 | C486T | Silent |
| British Shorthair | Fca2588 | A3Cc | 3 | T293A | W65R |
| British Shorthair | Fca2588 | A3Cc | 3 | C387G | F129L |
| British Shorthair | Fca2588 | A3Cc | 3 | A389G | Y130W |
| British Shorthair | Fca2588 | A3Cc | 3 | C390G | Y130W |
| British Shorthair | Fca2588 | A3Cc | 3 | C391G | H131D |
| British Shorthair | Fca2588 | A3Cc | 4 | C486T | Silent |
| Egyptian Mau | Fca137 | A3Cc | 3 | T293A | W65R^b^ |
| Egyptian Mau | Fca137 | A3Cc | 3 | C387G | F129L^b^ |
| Egyptian Mau | Fca137 | A3Cc | 4 | C486T | Silent^b^ |
| Norwegian Forest | Fca2156 | A3Cc | 2 | G63T | Silent^b^ |
| Norwegian Forest | Fca2156 | A3Cc | 3 | T293A | W65R^b^ |
| Norwegian Forest | Fca2156 | A3Cc | 3 | C387G | F129L^b^ |
| Norwegian Forest | Fca2156 | A3Cc | 3 | A389G | Y130W^b^ |
| Norwegian Forest | Fca2156 | A3Cc | 3 | C390G | Y130W^b^ |
| Norwegian Forest | Fca2156 | A3Cc | 3 | C391G | H131D^b^ |
| Norwegian Forest | Fca2156 | A3Cc | 4 | C486T | Silent^b^ |
| Sphynx | Fca1497 | A3Cc | 4 | C486T | Silent^b^ |
| Turkish van | Fca2417 | A3Cc | 3 | T293A | W65R^b^ |
| Turkish van | Fca2417 | A3Cc | 3 | C387G | F129L^b^ |
| Turkish van | Fca2417 | A3Cc | 3 | A389G | Y130W^b^ |
| Turkish van | Fca2417 | A3Cc | 3 | C390G | Y130W^b^ |
| Turkish van | Fca2417 | A3Cc | 3 | C391G | H131D^b^ |
| Turkish van | Fca2417 | A3Cc | 4 | C486T | Silent^b^ |

^a^ SNPS are numbered relative to the first base of the translational initiation codon

^b^ Validated by clonal sequencing

**Supplemental Table 5.** **Identities of A3C cDNAs and predicted proteins**

|  | ***Cat A3Ca Cat A3Cb Cat A3Cc Cat A3Ca Cat A3Cb Cat A3Cc***  ***id. nt. (%) id. aa. (%)*** | | | | | |
| --- | --- | --- | --- | --- | --- | --- |
| Puma A3C | 98.0 | 97.3 | 97.6 | 95.6 | 94.0 | 95.1 |
| Tiger A3C#1 | 95.3 | 95.3 | 95.3 | 91.2 | 90.7 | 90.1 |
| Tiger A3C#2 | 94.9 | 94.9 | 94.9 | 90.1 | 89.5 | 89.0 |
| Tiger A3C#3 | 95.3 | 95.3 | 95.3 | 91.2 | 90.7 | 90.1 |
| Lion A3C#1 | 94.0 | 94.0 | 95.1 | 85.7 | 85.2 | 87.9 |
| Lion A3C#2 | 94.5 | 94.5 | 94.5 | 88.5 | 87.9 | 87.4 |
| Leopard A3C | 94.0 | 94.0 | 95.1 | 85.7 | 85.2 | 87.9 |
| Lynx A3C#1 | 96.5 | 96.2 | 95.1 | 92.9 | 92.3 | 89.0 |
| Lynx A3C#2 | 94.9 | 94.5 | 94.2 | 89.6 | 89.0 | 87.4 |
| Lynx A3C#5 | 94.2 | 93.8 | 93.4 | 87.9 | 87.4 | 85.7 |
| Lynx A3C#6 | 96.5 | 96.2 | 95.1 | 92.3 | 91.8 | 88.5 |

Note: Sequences of the PCR primers were excluded.

**Supplemental Table 6. Identities of A3H cDNAs and predicted proteins**

|  | **Cat A3H**  ***id. nt. (%) id. aa. (%)*** | |
| --- | --- | --- |
| Puma A3H | 98.0 | 96.2 |
| Tiger A3H | 97.0 | 95.2 |
| Lion A3H | 96.8 | 94.7 |
| Leopard A3H | 96.8 | 94.7 |
| Lynx A3H | 97.8 | 95.7 |

Note: Sequences of the PCR primers were excluded.

**Supplemental Table 7.** **Results of different evolutionary models**

A: PAML result of the A3H tree of different Felids

| **Model used** | **Neg. log likelihood** | **Transition/transversion ratio** | **Ratio parameters** | **Omega values** | **Sites under pos. selection (NEB, pr>.99)** | **Sites under pos. selection (BEB, pr>0.9)** |
| --- | --- | --- | --- | --- | --- | --- |
| neutral(M1): | -844.994230 | kappa (ts/tv)=3.48054 | p: 0.41690 0.58310 | w: 0.00000 1.00000 |  |  |
| positive(M2): | -844.338667 | kappa (ts/tv)=3.78332 | p: 0.71526 0.00000 0.28474 | w: 0.00000 1.00000 2.77319 | 7 V, 10 C, 42 H, 56 M, 64 A, 89 E, 127 A, 151 Q, 154 S, 159 P, 160 N, 162 D, 166 K, 177 T |  |
| model7(beta): | -844.996538 | kappa (ts/tv)=3.50121 | p=0.00749  q=0.00500 |  |  |  |
| model8(beta,w>1): | -844.338751 | kappa (ts/tv)=3.78448 | p0=0.71747 p=0.45987 q=99.00000 | w=2.78214 | 7 V, 10 C, 42 H, 56 M, 64 A, 89 E, 127 A, 151 Q, 154 S, 159 P, 160 N, 162 D, 166 K, 177 T |  |

B: PAML results of the A3C tree of different Felids

| **Model used** | **Neg. log. likelihood** | **Transition/transversion ratio** | **Ratio parameters** | **Omega values** | **Sites under pos. selection (NEB, pr>.99)** | **Sites under pos. selection (BEB, pr>0.9)** |
| --- | --- | --- | --- | --- | --- | --- |
| neutral(M1): | -1420.997435 | kappa (ts/tv)=2.83247 | p: 0.45858 0.54142 | w: 0.00000 1.00000 |  |  |
| positive(M2): | -1405.413360 | kappa (ts/tv)=3.74936 | p: 0.77850 0.06323 0.15828 | w: 0.45723 1.00000 7.19982 | 18 N, 21 R, 55 R, 67 R, 130 W, 136 E, 139 C, 157 K, 158 H, 168 G, 174 R, 186 E | 18 N, 21 R, 130 W, 139 C |
| M7(beta): | -1421.131363 | kappa(ts/tv)=2.77448 | p=0.00502 q=0.00500 |  |  |  |
| M8(beta,w>1): | -1405.413679 | kappa (ts/tv)=3.74942 | p0=0.83983 p=32.96643 q=34.27576 | w=7.15846 | 18 N, 21 R, 55 R, 67 R, 130 W, 136 E, 139 C, 157 K, 158 H, 168 G, 174 R, 186 E | 18 N, 21 R, 130 W, 139 C |

Note: Only sites inferred under selection at the 95% level or above are listed. In case of A3C there are several additional sites at lower levels. The numbers of the sites correspond to the positions in the cat A3H protein or cat A3Ca protein. For the detailed description of the models, see [77, 78].
