# Supplementary material for: Functions, structure, and read-through alternative splicing of feline APOBEC3 genes
Source: Genome Biol. 2008 Mar 3;9(3):R48. doi: 10.1186/gb-2008-9-3-r48 (PMC2397500; doi:10.1186/gb-2008-9-3-r48)
Supplement: Additional data file 3 — Figure S1: comparison of amino acid sequences of the feline A3C genes. Predicted amino acid sequence of the feline APOBEC3Ca, APOBEC3Cb and APOBEC3Cc proteins in comparison with the two additional variant cDNAs detected (A3Cx and A3Cy) in cat PBMCs. The zinc coordination domain is indicated. Residues different to A3Ca are shown in bold. Figure S2: amino acid alignment of feline, canine and human APOBEC3 proteins. (a) Amino acid alignment of feline APOBEC3Ca, APOBEC3Cb, APOBEC3Cc, human APOBEC3C, APOBEC3F and murine APOBEC3 NT. (b) Amino acid alignment of feline, canine, human APOBEC3H and murine APOBEC3 NT. (c) Amino acid alignment of human, canine APOBEC3A and human APOBEC3G CT. The zinc-coordinating domains are indicated. CT, carboxyl-terminal domain; NT, amino-terminal domain. Figure S3: prediction of transcription factor binding sites. Potential transcription factor binding sites in A3 cluster of the domestic cat in the region 1.1 kb upstream, including 100 bp of the predicted exon 1 for each gene (A3Ca, A3Cb, A3Cc and A3H) using ClustalW. The individual 5' flanking sequences were analyzed using the program Match, which uses a library of nucleotide weight matrices from the TRANSFAC6.0 database for transcription factor binding sites. Figure S4: analysis of Ka/Ks. Sliding window (300 bp window, 50 bp slide) analysis of Ka and Ks was performed on pairs of (a) cat A3C sequences and (b) cat A3H sequences and compared with corresponding selected felid and human sequences. Ka/Ks is plotted against the length of the coding region of the mRNAs with a schematic presentation of protein domains along the x-axis. Figure S5: analysis of cytidine deamination in the genomes of FIV by feline APOBEC3s. (a) A fragment of the reporter gene (egfp) was amplified from reverse transcripts of Δvif FIV (left panel) or wild-type FIV (right panel) generated in the presence of the indicated feline APOBEC3s 10 h post-infection. A total of eight independent nucleotide sequences were determine [file gb-2008-9-3-r48-S3.ppt]

## Slide 1
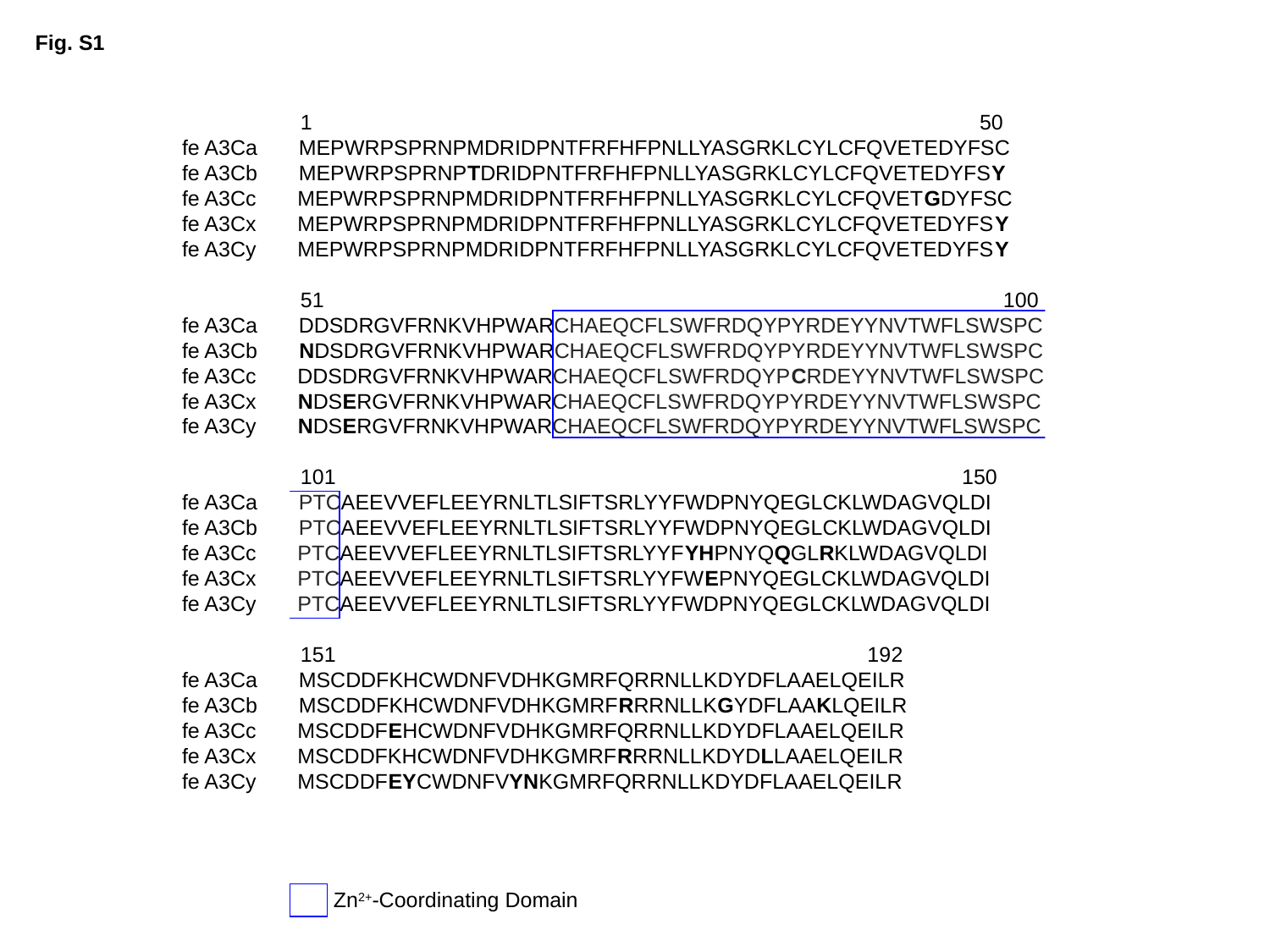

Fig. S1
 1 50
fe A3Ca MEPWRPSPRNPMDRIDPNTFRFHFPNLLYASGRKLCYLCFQVETEDYFSC
fe A3Cb MEPWRPSPRNPTDRIDPNTFRFHFPNLLYASGRKLCYLCFQVETEDYFSY
fe A3Cc MEPWRPSPRNPMDRIDPNTFRFHFPNLLYASGRKLCYLCFQVETGDYFSC
fe A3Cx MEPWRPSPRNPMDRIDPNTFRFHFPNLLYASGRKLCYLCFQVETEDYFSY
fe A3Cy MEPWRPSPRNPMDRIDPNTFRFHFPNLLYASGRKLCYLCFQVETEDYFSY
 51 100
fe A3Ca DDSDRGVFRNKVHPWARCHAEQCFLSWFRDQYPYRDEYYNVTWFLSWSPC
fe A3Cb NDSDRGVFRNKVHPWARCHAEQCFLSWFRDQYPYRDEYYNVTWFLSWSPC
fe A3Cc DDSDRGVFRNKVHPWARCHAEQCFLSWFRDQYPCRDEYYNVTWFLSWSPC
fe A3Cx NDSERGVFRNKVHPWARCHAEQCFLSWFRDQYPYRDEYYNVTWFLSWSPC
fe A3Cy NDSERGVFRNKVHPWARCHAEQCFLSWFRDQYPYRDEYYNVTWFLSWSPC
 101 150
fe A3Ca PTCAEEVVEFLEEYRNLTLSIFTSRLYYFWDPNYQEGLCKLWDAGVQLDI
fe A3Cb PTCAEEVVEFLEEYRNLTLSIFTSRLYYFWDPNYQEGLCKLWDAGVQLDI
fe A3Cc PTCAEEVVEFLEEYRNLTLSIFTSRLYYFYHPNYQQGLRKLWDAGVQLDI
fe A3Cx PTCAEEVVEFLEEYRNLTLSIFTSRLYYFWEPNYQEGLCKLWDAGVQLDI
fe A3Cy PTCAEEVVEFLEEYRNLTLSIFTSRLYYFWDPNYQEGLCKLWDAGVQLDI
 151 192
fe A3Ca MSCDDFKHCWDNFVDHKGMRFQRRNLLKDYDFLAAELQEILR
fe A3Cb MSCDDFKHCWDNFVDHKGMRFRRRNLLKGYDFLAAKLQEILR
fe A3Cc MSCDDFEHCWDNFVDHKGMRFQRRNLLKDYDFLAAELQEILR
fe A3Cx MSCDDFKHCWDNFVDHKGMRFRRRNLLKDYDLLAAELQEILR
fe A3Cy MSCDDFEYCWDNFVYNKGMRFQRRNLLKDYDFLAAELQEILR
Zn2+-Coordinating Domain

## Slide 2
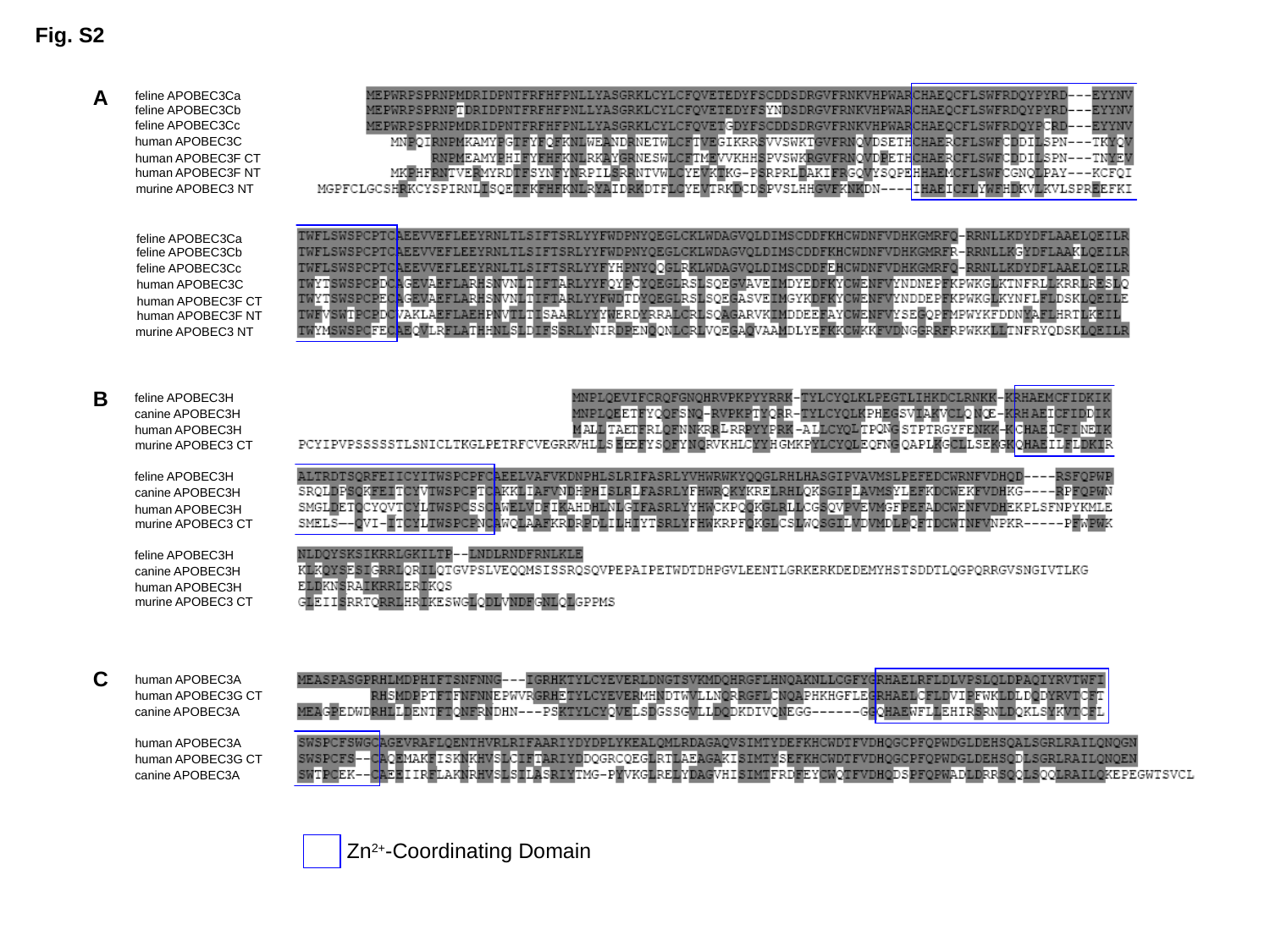

Fig. S2
A
feline APOBEC3Ca
feline APOBEC3Cb
feline APOBEC3Cc
human APOBEC3C
human APOBEC3F CT
human APOBEC3F NT
murine APOBEC3 NT
feline APOBEC3Ca
feline APOBEC3Cb
feline APOBEC3Cc
human APOBEC3C
human APOBEC3F CT
human APOBEC3F NT
murine APOBEC3 NT
B
feline APOBEC3H
canine APOBEC3H
human APOBEC3H
murine APOBEC3 CT
feline APOBEC3H
canine APOBEC3H
human APOBEC3H
murine APOBEC3 CT
feline APOBEC3H
canine APOBEC3H
human APOBEC3H
murine APOBEC3 CT
C
human APOBEC3A
human APOBEC3G CT
canine APOBEC3A
human APOBEC3A
human APOBEC3G CT
canine APOBEC3A
Zn2+-Coordinating Domain

## Slide 3
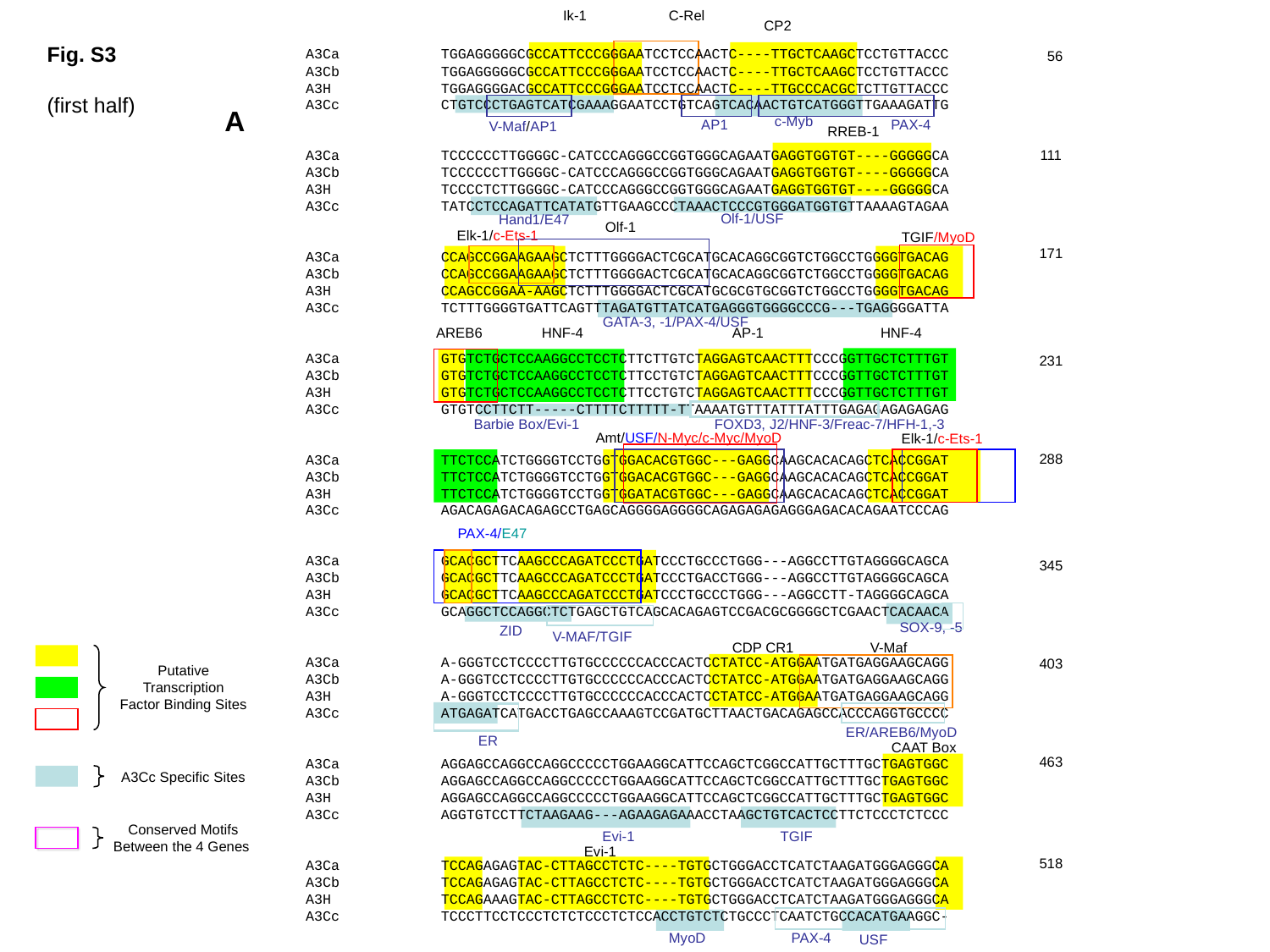

Ik-1
C-Rel
CP2
Fig. S3
(first half)
A3Ca TGGAGGGGGCGCCATTCCCGGGAATCCTCCAACTC----TTGCTCAAGCTCCTGTTACCC
A3Cb TGGAGGGGGCGCCATTCCCGGGAATCCTCCAACTC----TTGCTCAAGCTCCTGTTACCC
A3H TGGAGGGGACGCCATTCCCGGGAATCCTCCAACTC----TTGCCCACGCTCTTGTTACCC
A3Cc CTGTCCCTGAGTCATCGAAAGGAATCCTGTCAGTCACAACTGTCATGGGTTGAAAGATTG
A3Ca TCCCCCCTTGGGGC-CATCCCAGGGCCGGTGGGCAGAATGAGGTGGTGT----GGGGGCA
A3Cb TCCCCCCTTGGGGC-CATCCCAGGGCCGGTGGGCAGAATGAGGTGGTGT----GGGGGCA
A3H TCCCCTCTTGGGGC-CATCCCAGGGCCGGTGGGCAGAATGAGGTGGTGT----GGGGGCA
A3Cc TATCCTCCAGATTCATATGTTGAAGCCCTAAACTCCCGTGGGATGGTGTTAAAAGTAGAA
A3Ca CCAGCCGGAAGAAGCTCTTTGGGGACTCGCATGCACAGGCGGTCTGGCCTGGGGTGACAG
A3Cb CCAGCCGGAAGAAGCTCTTTGGGGACTCGCATGCACAGGCGGTCTGGCCTGGGGTGACAG
A3H CCAGCCGGAA-AAGCTCTTTGGGGACTCGCATGCGCGTGCGGTCTGGCCTGGGGTGACAG
A3Cc TCTTTGGGGTGATTCAGTTTAGATGTTATCATGAGGGTGGGGCCCG---TGAGGGGATTA
A3Ca GTGTCTGCTCCAAGGCCTCCTCTTCTTGTCTAGGAGTCAACTTTCCCGGTTGCTCTTTGT
A3Cb GTGTCTGCTCCAAGGCCTCCTCTTCCTGTCTAGGAGTCAACTTTCCCGGTTGCTCTTTGT
A3H GTGTCTGCTCCAAGGCCTCCTCTTCCTGTCTAGGAGTCAACTTTCCCGGTTGCTCTTTGT
A3Cc GTGTCCTTCTT-----CTTTTCTTTTT-TTAAAATGTTTATTTATTTGAGAGAGAGAGAG
A3Ca TTCTCCATCTGGGGTCCTGGTGGACACGTGGC---GAGGCAAGCACACAGCTCACCGGAT
A3Cb TTCTCCATCTGGGGTCCTGGTGGACACGTGGC---GAGGCAAGCACACAGCTCACCGGAT
A3H TTCTCCATCTGGGGTCCTGGTGGATACGTGGC---GAGGCAAGCACACAGCTCACCGGAT
A3Cc AGACAGAGACAGAGCCTGAGCAGGGGAGGGGCAGAGAGAGAGGGAGACACAGAATCCCAG
A3Ca GCACGCTTCAAGCCCAGATCCCTGATCCCTGCCCTGGG---AGGCCTTGTAGGGGCAGCA
A3Cb GCACGCTTCAAGCCCAGATCCCTGATCCCTGACCTGGG---AGGCCTTGTAGGGGCAGCA
A3H GCACGCTTCAAGCCCAGATCCCTGATCCCTGCCCTGGG---AGGCCTT-TAGGGGCAGCA
A3Cc GCAGGCTCCAGGCTCTGAGCTGTCAGCACAGAGTCCGACGCGGGGCTCGAACTCACAACA
A3Ca A-GGGTCCTCCCCTTGTGCCCCCCACCCACTCCTATCC-ATGGAATGATGAGGAAGCAGG
A3Cb A-GGGTCCTCCCCTTGTGCCCCCCACCCACTCCTATCC-ATGGAATGATGAGGAAGCAGG
A3H A-GGGTCCTCCCCTTGTGCCCCCCACCCACTCCTATCC-ATGGAATGATGAGGAAGCAGG
A3Cc ATGAGATCATGACCTGAGCCAAAGTCCGATGCTTAACTGACAGAGCCACCCAGGTGCCCC
A3Ca AGGAGCCAGGCCAGGCCCCCTGGAAGGCATTCCAGCTCGGCCATTGCTTTGCTGAGTGGC
A3Cb AGGAGCCAGGCCAGGCCCCCTGGAAGGCATTCCAGCTCGGCCATTGCTTTGCTGAGTGGC
A3H AGGAGCCAGGCCAGGCCCCCTGGAAGGCATTCCAGCTCGGCCATTGCTTTGCTGAGTGGC
A3Cc AGGTGTCCTTCTAAGAAG---AGAAGAGAAACCTAAGCTGTCACTCCTTCTCCCTCTCCC
A3Ca TCCAGAGAGTAC-CTTAGCCTCTC----TGTGCTGGGACCTCATCTAAGATGGGAGGGCA
A3Cb TCCAGAGAGTAC-CTTAGCCTCTC----TGTGCTGGGACCTCATCTAAGATGGGAGGGCA
A3H TCCAGAAAGTAC-CTTAGCCTCTC----TGTGCTGGGACCTCATCTAAGATGGGAGGGCA
A3Cc TCCCTTCCTCCCTCTCTCCCTCTCCACCTGTCTCTGCCCTCAATCTGCCACATGAAGGC-
56
A
c-Myb
AP1
PAX-4
V-Maf/AP1
RREB-1
111
Olf-1/USF
Hand1/E47
Olf-1
Elk-1/c-Ets-1
TGIF/MyoD
171
GATA-3, -1/PAX-4/USF
AREB6
HNF-4
AP-1
HNF-4
231
Barbie Box/Evi-1
FOXD3, J2/HNF-3/Freac-7/HFH-1,-3
Amt/USF/N-Myc/c-Myc/MyoD
Elk-1/c-Ets-1
288
PAX-4/E47
345
SOX-9, -5
ZID
V-MAF/TGIF
CDP CR1
V-Maf
Putative
Transcription
Factor Binding Sites
A3Cc Specific Sites
Conserved Motifs
Between the 4 Genes
403
ER/AREB6/MyoD
ER
CAAT Box
463
Evi-1
TGIF
Evi-1
518
MyoD
PAX-4
USF

## Slide 4
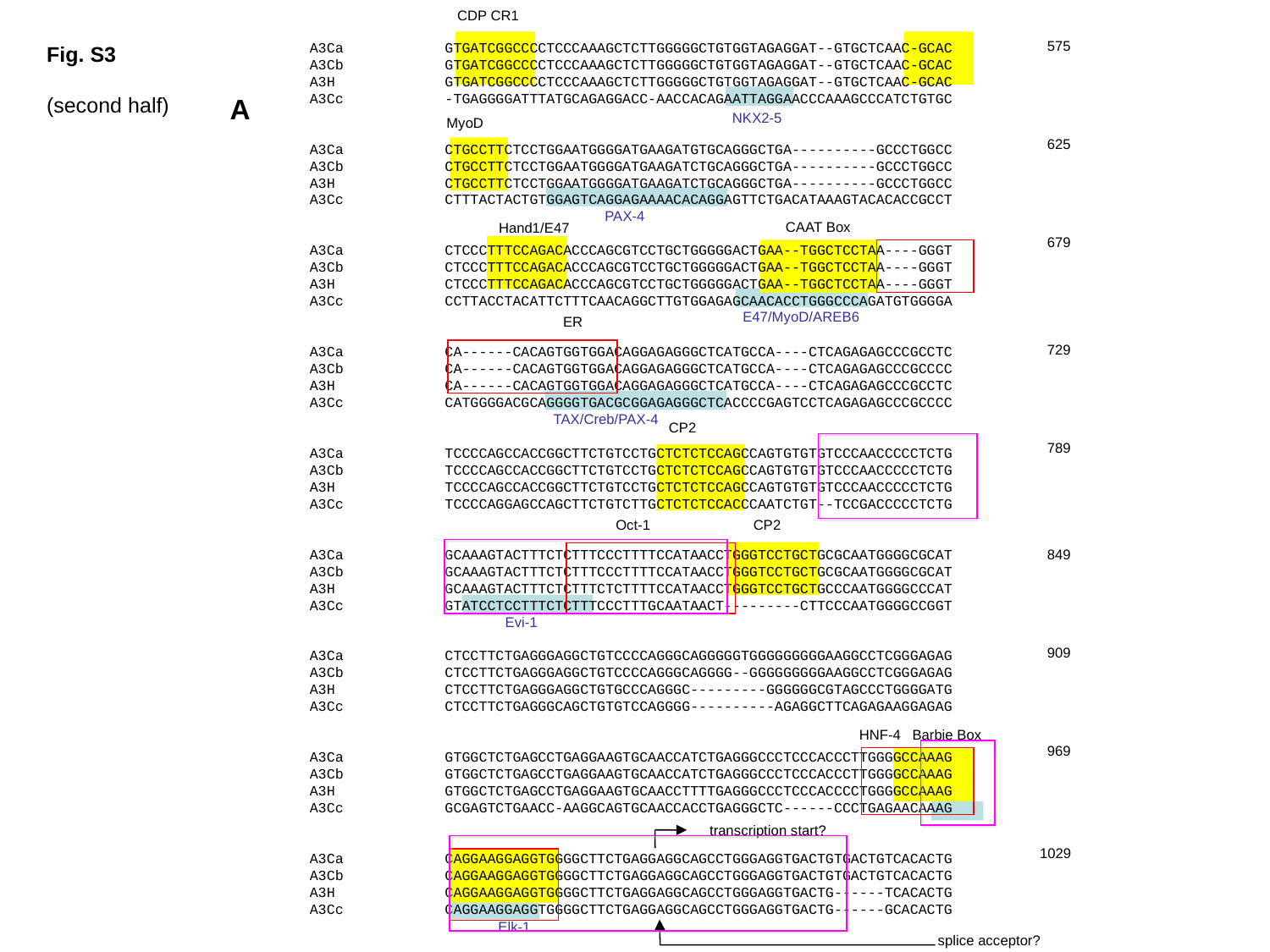

CDP CR1
A3Ca GTGATCGGCCCCTCCCAAAGCTCTTGGGGGCTGTGGTAGAGGAT--GTGCTCAAC-GCAC
A3Cb GTGATCGGCCCCTCCCAAAGCTCTTGGGGGCTGTGGTAGAGGAT--GTGCTCAAC-GCAC
A3H GTGATCGGCCCCTCCCAAAGCTCTTGGGGGCTGTGGTAGAGGAT--GTGCTCAAC-GCAC
A3Cc -TGAGGGGATTTATGCAGAGGACC-AACCACAGAATTAGGAACCCAAAGCCCATCTGTGC
A3Ca CTGCCTTCTCCTGGAATGGGGATGAAGATGTGCAGGGCTGA----------GCCCTGGCC
A3Cb CTGCCTTCTCCTGGAATGGGGATGAAGATCTGCAGGGCTGA----------GCCCTGGCC
A3H CTGCCTTCTCCTGGAATGGGGATGAAGATCTGCAGGGCTGA----------GCCCTGGCC
A3Cc CTTTACTACTGTGGAGTCAGGAGAAAACACAGGAGTTCTGACATAAAGTACACACCGCCT
A3Ca CTCCCTTTCCAGACACCCAGCGTCCTGCTGGGGGACTGAA--TGGCTCCTAA----GGGT
A3Cb CTCCCTTTCCAGACACCCAGCGTCCTGCTGGGGGACTGAA--TGGCTCCTAA----GGGT
A3H CTCCCTTTCCAGACACCCAGCGTCCTGCTGGGGGACTGAA--TGGCTCCTAA----GGGT
A3Cc CCTTACCTACATTCTTTCAACAGGCTTGTGGAGAGCAACACCTGGGCCCAGATGTGGGGA
A3Ca CA------CACAGTGGTGGACAGGAGAGGGCTCATGCCA----CTCAGAGAGCCCGCCTC
A3Cb CA------CACAGTGGTGGACAGGAGAGGGCTCATGCCA----CTCAGAGAGCCCGCCCC
A3H CA------CACAGTGGTGGACAGGAGAGGGCTCATGCCA----CTCAGAGAGCCCGCCTC
A3Cc CATGGGGACGCAGGGGTGACGCGGAGAGGGCTCACCCCGAGTCCTCAGAGAGCCCGCCCC
A3Ca TCCCCAGCCACCGGCTTCTGTCCTGCTCTCTCCAGCCAGTGTGTGTCCCAACCCCCTCTG
A3Cb TCCCCAGCCACCGGCTTCTGTCCTGCTCTCTCCAGCCAGTGTGTGTCCCAACCCCCTCTG
A3H TCCCCAGCCACCGGCTTCTGTCCTGCTCTCTCCAGCCAGTGTGTGTCCCAACCCCCTCTG
A3Cc TCCCCAGGAGCCAGCTTCTGTCTTGCTCTCTCCACCCAATCTGT--TCCGACCCCCTCTG
A3Ca GCAAAGTACTTTCTCTTTCCCTTTTCCATAACCTGGGTCCTGCTGCGCAATGGGGCGCAT
A3Cb GCAAAGTACTTTCTCTTTCCCTTTTCCATAACCTGGGTCCTGCTGCGCAATGGGGCGCAT
A3H GCAAAGTACTTTCTCTTTCTCTTTTCCATAACCTGGGTCCTGCTGCCCAATGGGGCCCAT
A3Cc GTATCCTCCTTTCTCTTTCCCTTTGCAATAACT---------CTTCCCAATGGGGCCGGT
A3Ca CTCCTTCTGAGGGAGGCTGTCCCCAGGGCAGGGGGTGGGGGGGGGAAGGCCTCGGGAGAG
A3Cb CTCCTTCTGAGGGAGGCTGTCCCCAGGGCAGGGG--GGGGGGGGGAAGGCCTCGGGAGAG
A3H CTCCTTCTGAGGGAGGCTGTGCCCAGGGC---------GGGGGGCGTAGCCCTGGGGATG
A3Cc CTCCTTCTGAGGGCAGCTGTGTCCAGGGG----------AGAGGCTTCAGAGAAGGAGAG
A3Ca GTGGCTCTGAGCCTGAGGAAGTGCAACCATCTGAGGGCCCTCCCACCCTTGGGGCCAAAG
A3Cb GTGGCTCTGAGCCTGAGGAAGTGCAACCATCTGAGGGCCCTCCCACCCTTGGGGCCAAAG
A3H GTGGCTCTGAGCCTGAGGAAGTGCAACCTTTTGAGGGCCCTCCCACCCCTGGGGCCAAAG
A3Cc GCGAGTCTGAACC-AAGGCAGTGCAACCACCTGAGGGCTC------CCCTGAGAACAAAG
A3Ca CAGGAAGGAGGTGGGGCTTCTGAGGAGGCAGCCTGGGAGGTGACTGTGACTGTCACACTG
A3Cb CAGGAAGGAGGTGGGGCTTCTGAGGAGGCAGCCTGGGAGGTGACTGTGACTGTCACACTG
A3H CAGGAAGGAGGTGGGGCTTCTGAGGAGGCAGCCTGGGAGGTGACTG------TCACACTG
A3Cc CAGGAAGGAGGTGGGGCTTCTGAGGAGGCAGCCTGGGAGGTGACTG------GCACACTG
575
Fig. S3
(second half)
A
NKX2-5
MyoD
625
PAX-4
CAAT Box
Hand1/E47
679
E47/MyoD/AREB6
ER
729
TAX/Creb/PAX-4
CP2
789
Oct-1
CP2
849
Evi-1
909
HNF-4
Barbie Box
969
transcription start?
1029
Elk-1
splice acceptor?

## Slide 5
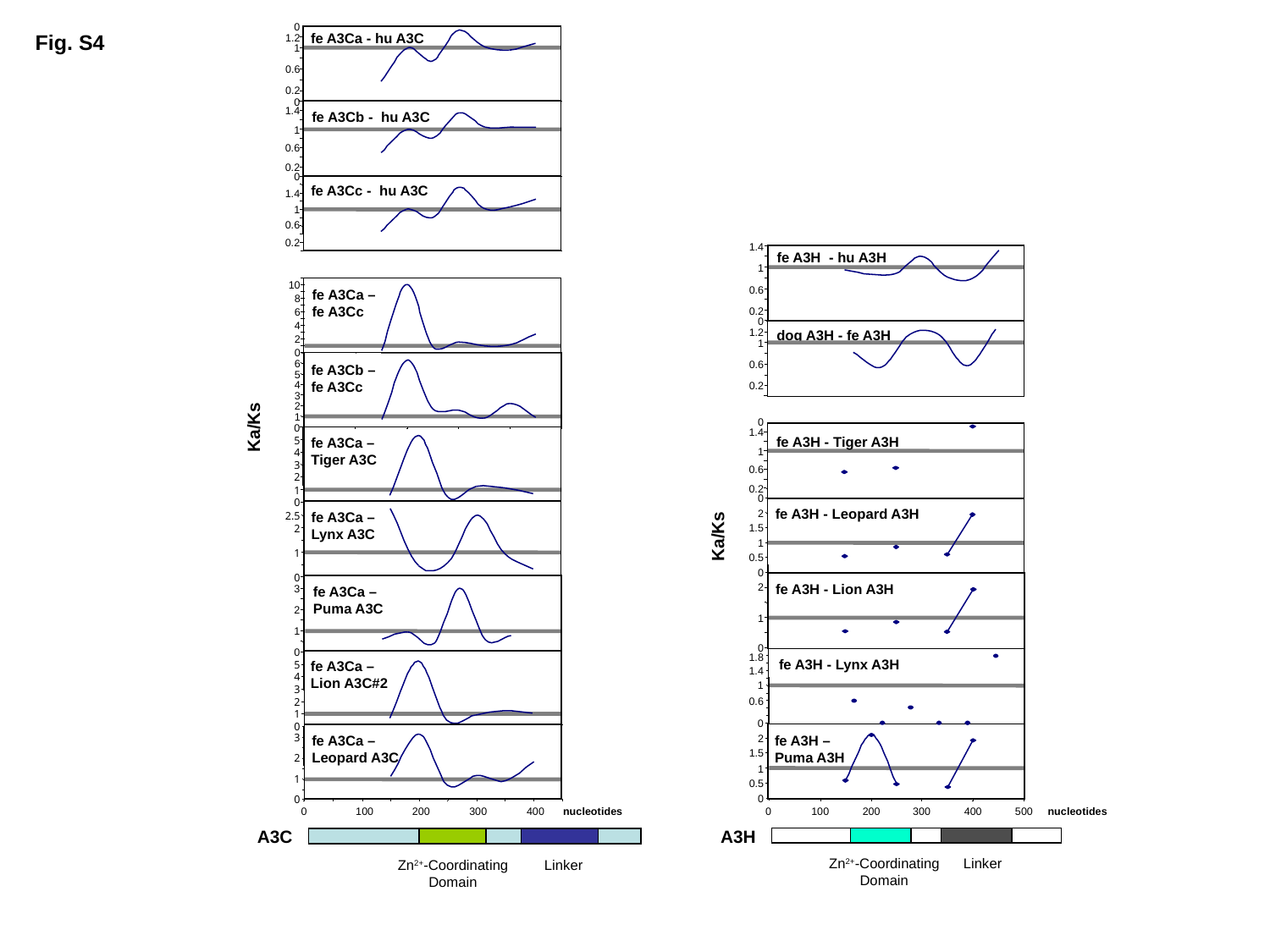

0
fe A3Ca - hu A3C
1.2
1
0.6
0.2
0
1.4
fe A3Cb - hu A3C
1
0.6
0.2
0
fe A3Cc - hu A3C
1.4
1
0.6
0.2
Fig. S4
1.4
fe A3H - hu A3H
1
0.6
0.2
0
1.2
dog A3H - fe A3H
1
0.6
0.2
10
fe A3Ca –
fe A3Cc
8
6
4
2
0
6
fe A3Cb –
fe A3Cc
5
4
3
2
1
0
Ka/Ks
0
1.4
fe A3H - Tiger A3H
5
fe A3Ca –
Tiger A3C
1
4
3
0.6
2
0.2
1
0
0
fe A3H - Leopard A3H
2
fe A3Ca –
Lynx A3C
2.5
1.5
2
Ka/Ks
1
1
0.5
0
0
fe A3H - Lion A3H
2
3
fe A3Ca –
Puma A3C
2
1
1
0
0
1.8
fe A3H - Lynx A3H
fe A3Ca –
Lion A3C#2
5
1.4
4
1
3
0.6
2
1
0
0
3
fe A3H –
Puma A3H
fe A3Ca –
Leopard A3C
2
1.5
2
1
1
0.5
0
0
0
100
200
300
400
nucleotides
0
100
200
300
400
500
nucleotides
A3H
A3C
Zn2+-Coordinating
Domain
Linker
Zn2+-Coordinating
Domain
Linker

## Slide 6
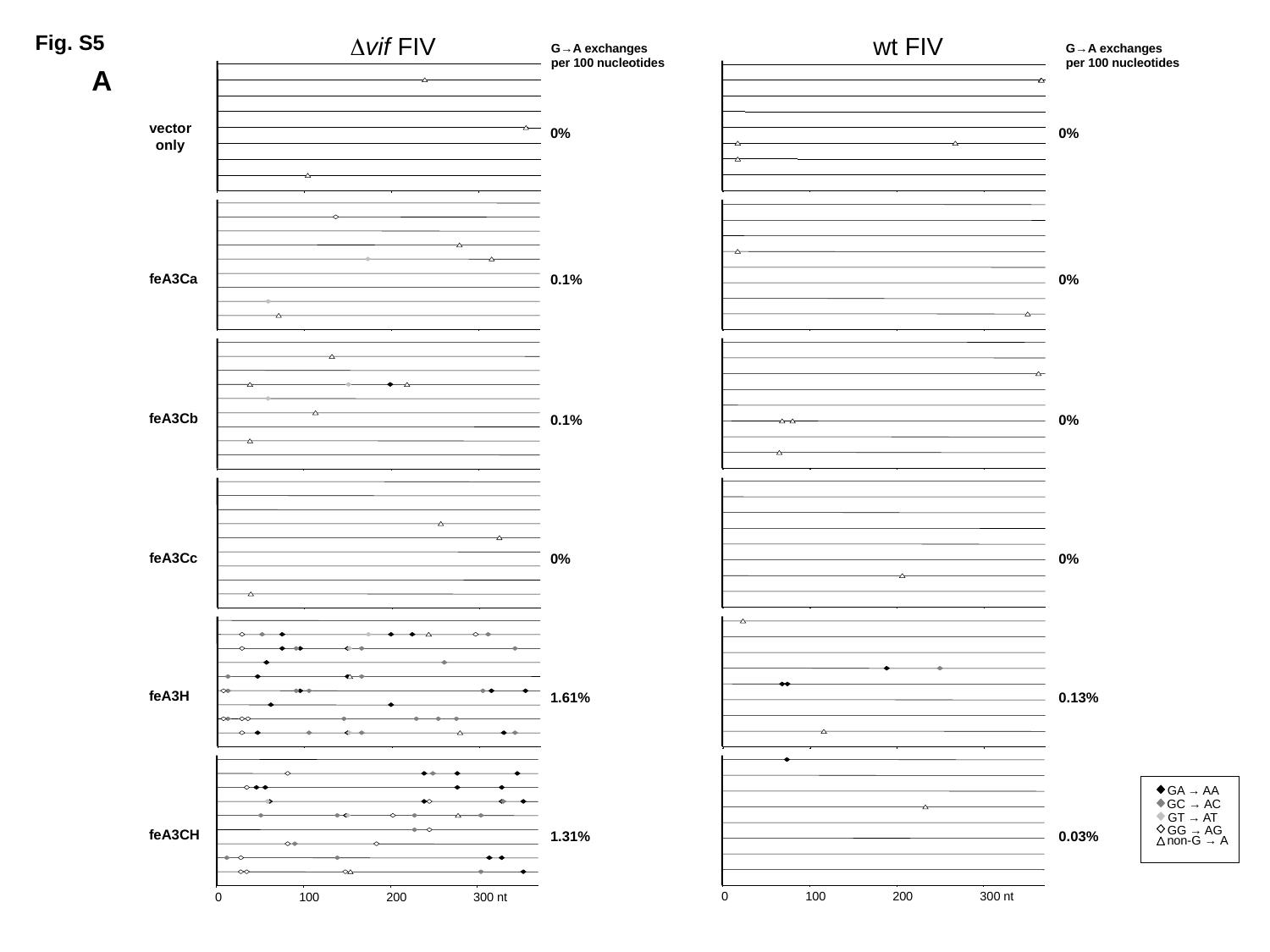

Fig. S5
Δvif FIV
wt FIV
G→A exchanges
per 100 nucleotides
G→A exchanges
per 100 nucleotides
A
vector
only
0%
0%
feA3Ca
0.1%
0%
feA3Cb
0.1%
0%
feA3Cc
0%
0%
feA3H
1.61%
0.13%
0
100
200
300 nt
0
100
200
300 nt
GA → AA
GC → AC
GT → AT
feA3CH
1.31%
0.03%
GG → AG
non-G → A

## Slide 7
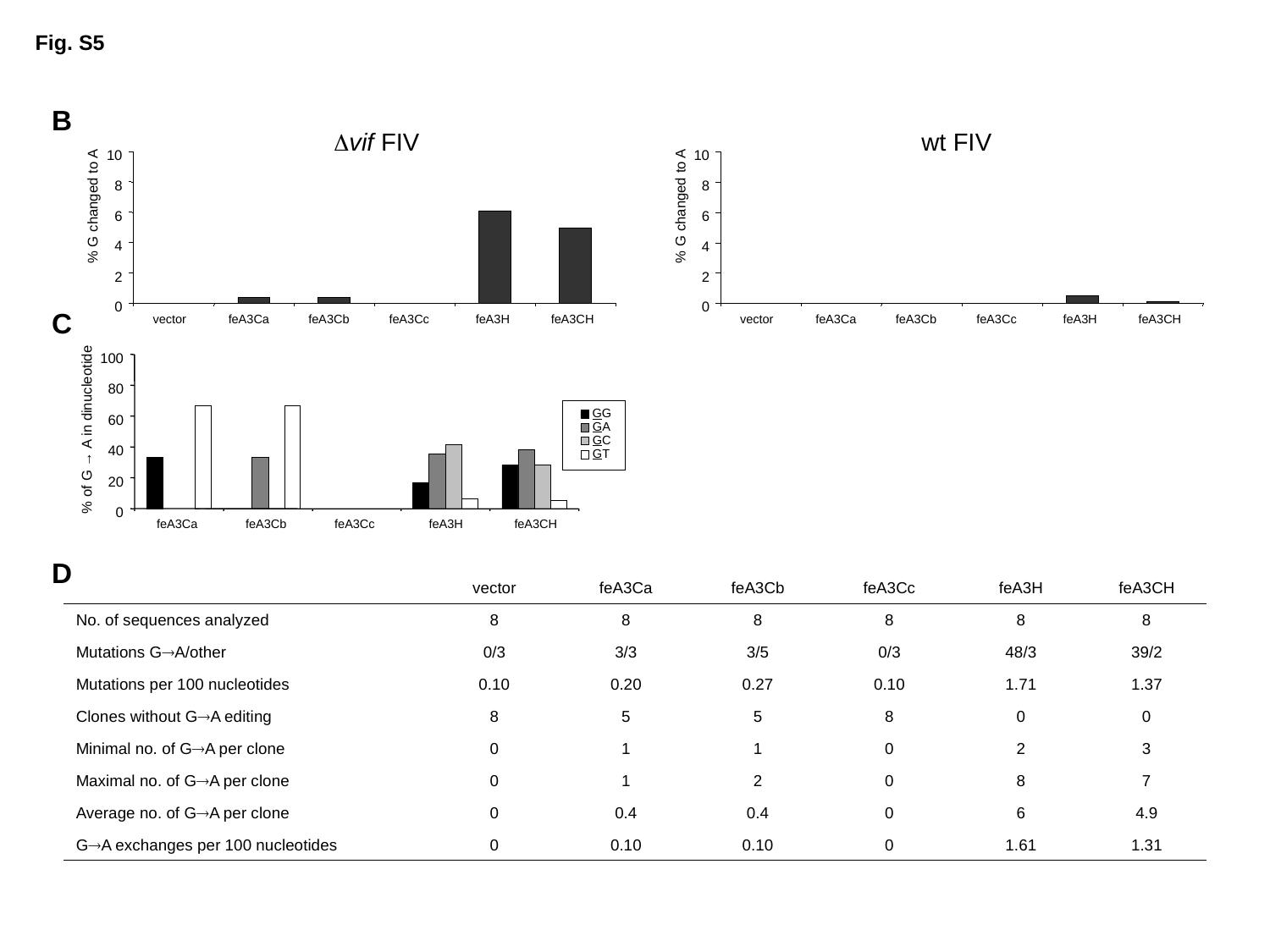

Fig. S5
B
wt FIV
Δvif FIV
10
10
8
8
% G changed to A
% G changed to A
6
6
4
4
2
2
0
0
C
vector
feA3Ca
feA3Cb
feA3Cc
feA3H
feA3CH
vector
feA3Ca
feA3Cb
feA3Cc
feA3H
feA3CH
100
80
GG
60
GA
% of G → A in dinucleotide
GC
40
GT
20
0
feA3Ca
feA3Cb
feA3Cc
feA3H
feA3CH
D
| | | | vector | feA3Ca | feA3Cb | feA3Cc | feA3H | feA3CH |
| --- | --- | --- | --- | --- | --- | --- | --- | --- |
| No. of sequences analyzed | | | 8 | 8 | 8 | 8 | 8 | 8 |
| Mutations GA/other | | | 0/3 | 3/3 | 3/5 | 0/3 | 48/3 | 39/2 |
| Mutations per 100 nucleotides | | | 0.10 | 0.20 | 0.27 | 0.10 | 1.71 | 1.37 |
| Clones without GA editing | | | 8 | 5 | 5 | 8 | 0 | 0 |
| Minimal no. of GA per clone | | | 0 | 1 | 1 | 0 | 2 | 3 |
| Maximal no. of GA per clone | | | 0 | 1 | 2 | 0 | 8 | 7 |
| Average no. of GA per clone | | | 0 | 0.4 | 0.4 | 0 | 6 | 4.9 |
| GA exchanges per 100 nucleotides | | | 0 | 0.10 | 0.10 | 0 | 1.61 | 1.31 |
